# Splitting statistical potentials into meaningful scoring functions: Testing the prediction of near-native structures from decoy conformations

**DOI:** 10.1186/1472-6807-9-71

**Published:** 2009-11-16

**Authors:** Patrick Aloy, Baldo Oliva

**Affiliations:** 1 Institut de Recerca Biomèdica (IRB) and Barcelona Supercomputing Center (BSC), c/Baldiri i Reixac, 10-12 08028 Barcelona, Catalonia, Spain; 2 Institució Catalana de Recerca i Estudis Avançats (ICREA), Passeig Lluís Companys, 23 08010-Barcelona, Catalonia, Spain; 3 Structural Bioinformatics Group, (GRIB-IMIM), Departament de Ciències Eperimentals i de la Salut, Universitat Pompeu Fabra, Barcelona Research Park of Biomedicine (PRBB), 08003-Barcelona, Catalonia, Spain

## Abstract

**Background:**

Recent advances on high-throughput technologies have produced a vast amount of protein sequences, while the number of high-resolution structures has seen a limited increase. This has impelled the production of many strategies to built protein structures from its sequence, generating a considerable amount of alternative models. The selection of the closest model to the native conformation has thus become crucial for structure prediction. Several methods have been developed to score protein models by energies, knowledge-based potentials and combination of both.

**Results:**

Here, we present and demonstrate a theory to split the knowledge-based potentials in scoring terms biologically meaningful and to combine them in new scores to predict near-native structures. Our strategy allows circumventing the problem of defining the reference state. In this approach we give the proof for a simple and linear application that can be further improved by optimizing the combination of Zscores. Using the simplest composite score () we obtained predictions similar to state-of-the-art methods. Besides, our approach has the advantage of identifying the most relevant terms involved in the stability of the protein structure. Finally, we also use the composite Zscores to assess the conformation of models and to detect local errors.

**Conclusion:**

We have introduced a method to split knowledge-based potentials and to solve the problem of defining a reference state. The new scores have detected near-native structures as accurately as state-of-art methods and have been successful to identify wrongly modeled regions of many near-native conformations.

## Background

The study of the conformational space explored by a protein has long been of interest to structural biologists. The small region of this conformational space in which a protein is biologically active is known as its native state. The native state generally has the lowest free energy of all states under the native conditions [1], and the physical mechanism by which a protein finds it is known as the folding pathway. The vastness of the search space for a folding protein was first appreciated by Levinthal [2] who conceived the paradox of a long and non-biological time scale needed for a folding mechanism based on random pathways [3]. The solution of the protein folding problem requires an accurate potential that describes the interactions among different amino acid residues to enable the prediction and assessment of protein structures [4,5]. However, the use of such physical-based potentials [6,7] is computationally prohibitive and often it cannot ensure the native and biologically active conformation. Therefore, an alternative approach to the full atomistic description was to construct a scoring function whose global minimum corresponded to the native structure [8,9]. This scoring function is obtained by analysing the set of known native high-resolution structures deposited in the Protein Data Bank (PDB) [10] and it is termed as knowledge-based or statistical potential.

State-of the art methods are often able to predict the three-dimensional (3D) structure of protein domains with a RMSD (root mean square deviation) from native conformation ranging between 1Å and 6Å, where models with RMSD smaller than 2Å imply a resolution comparable to many experimentally obtained structures[11]. Among these methods, fold recognition and comparative modeling belong to the category of template-based modelling while *de novo *methods do not rely on any similarity on the fold level to known 3D structures (template-free) [12]. State of the art of structure prediction procedures (e.g. MODELLER [13], SWISS-MODEL[14], 3D-JIGSAW [15] for comparative modelling 3D-PSSM/PHYRE [16,17], TOPITS [18], GenTHREADER [19], LOOPP [20], FUGUE [21] for fold recognition, or TASSER [22], ROSETTA [23], PCONS [24], 3D-SHOTGUN [25], CABS [24] for *de novo *prediction [26]) are able to assemble approximately correct structures when a weakly homologous structure is available in the PDB [27]. However, the main problem displayed by most methods is the impossibility to distinguish a correct (i.e. near-native) model from a plethora of generated solutions. Selecting the closest model to the native conformation of a given protein out of an ensemble of models [28-30] is thus the crucial step for the protein structure prediction [12].

There are some common problems shared between template-based *de novo *prediction methods related to the selection of templates, detection of errors, and refinement of structures. For instance, one needs an energy function whose global minimum is in the protein's native state and which energy surface is funnel-like to drive the structure toward native-like conformations (i.e. having a correlation with native structure similarity [5,11]). These conditions have led many authors to use specialized scoring functions [12,31,32] and to combine knowledge-based force-fields and physical force fields with different objectives: 1) assessment of the correct fold [33]; 2) detection of local errors after modelling [34]; 3) studying the stability of mutant proteins [35,36]; discriminating between native and near-native states [32,37,38]; and 4) selecting near-native conformations in a set of decoys without the native structure [31,39].

On the one hand, statistical potentials have been derived for structural features such as torsion angles [12] and solvent accessibility [40]. In addition, residue-residue and all-atom based statistical potentials can be categorized into distance-independent contact energies [41] and distance-dependent potentials [32,42,43]. Furthermore, statistical potentials for the all-atom representation are generally more accurate than those that represent the interaction with centroids of amino-acid residues [44-46]. A vast amount of statistical potentials have been described and tested (see [32] for a detailed list). Many works have focused on the combination of knowledge-based potentials using artificial intelligence (i.e *GA*_*341 *_score obtained with a genetic algorithm [45], *ProQ *[47] and *GenThreader *[19] scores derived with artificial neural networks, composite score using support vector machines (SVM) regression[38]) and some have included physics-based energy functions with atomic detailed description of the interactions[46,48], like hydrophobic[36,49], hydrogen bonding, electrostatic, van der Waals, backbone torsions and binding harmonic terms (i.e. *QMEAN *[12], a funnel-like shape for the *Amber ff03-based *potential [5,11,50], or *FoldX *that uses a linear combination of energy components[51]). These approaches have prompted the problem lying on the physics of knowledge-based potentials: 1) what is the origin of the Boltzmann-like distribution for structural features in a sample of native structures [52]; 2) what is the most appropriate reference state [53]?; 3) is it possible the addition of individual terms of a statistical potential [32]?; 4) what is the offset between statistical potential(s) and other energetic terms to define a scoring function that predicts protein structure [54]?; and 5) what's the connection between statistic potentials and the energy-landscape of the free energy of a protein?. On the first two questions, the origin of the Boltzmann-like contribution and the definition of the reference state are still controversial. On the third and last question, Simons et al. presented a detailed derivation of scoring functions with particular attention to the interplay between solvation and residue pair interactions to split the terms involved in the statistical potential[55,56]. They provided a recipe for combining environment and residue pair specific effects in a systematic and non-redundant manner in *ROSETTA*[23]. Although the addition of the components of the energy cannot be transformed in the addition of free energy terms [57], it is still possible to split in different features the knowledge-based potential and to include additional terms on the core of a scoring function [55,56]. This permitted the evaluation of effectiveness in recognizing native-like structures among large decoy sets using different descriptions of sequence-dependent and sequence-independent features of proteins (i.e. remarking the relevance of including terms that describe the packing of *β*-strands in *β*-sheets) [56].

In this work we demonstrate the decomposition of knowledge-based potentials in energy terms with different levels of detail of residue-residue interactions. The new potential is based on the sum of terms that describe sequence-dependent/independent and distance-dependent/independent features of proteins that show biological and functional significance (i.e. remarking a specific environment for a particular residue). Our approach also circumvents the problem of a reference state of the statistical potential by means of a spare function without relevance on the assessment of native conformation. Finally, we compare our composite scoring function to other knowledge-based functions on: i) characterizing the relevance of the potential terms involved in native and near-native conformations; ii) finding the native conformation of several target proteins among decoy structures; iii) detecting near-native conformations; and iv) identifying local conformational errors.

## Outline of the algorithm

Our goal is to develop a new scoring potential independent of a reference state, able to discriminate between native and non-native conformations of proteins and able to detect local errors of a protein structure. This was obtained by: i) decomposing the score function in terms where some of them were functions of the reference state; ii) transforming the score into a sum of Zscores where the Zscore of the functions containing the reference state could be neglected; and iii) proving that the Zscore definition could still be applied to score the accommodation of individual residues in the structure. Here we present an outline of the algorithm. Details of the development of the equations are in the additional files (see Additional file 1: Supplemental of theory).

The interaction between two residues can be described by means of a potential of mean force[58,59]. Energy can usually be split in independent terms from which different forces are derived. Therefore, we also wish to split the statistical potential in terms that would describe the different parts of the interaction. The disconnection of energetic terms can be used not only to recognize the main interactions, but also to improve its individual expectation-values compared with a random approach. Our approach is similar to the scoring method in ROSETTA by Simons et al. [55,56], where local and structural environment play an important role with the sequence.

A potential of mean force has usually been used to score the interaction between two residues. The distance between a pair of residues can be calculated as the minimum distance between all atoms of both residues or as the distance between the C*β *atoms (C*α *for Glycine residues). The maximum distance to calculate the potential of mean force is different depending on this definition (i.e. 5Å for the minimum distance and 12Å for C*β*-C*β *distance). Force fields obtained with C*β*-C*β *distances are named C*β*-C*β *force-fields or C*β*-potentials, while those obtained with minimum distances are named *min *force-fields or *min*-potentials.

We have defined a new set of knowledge-based potential terms converting the reference state function into a new energy component. The new score is defined in equation 1 and derived by comparison with the standard definition of knowledge-based potential (see Additional file 1: Supplemental on theory)(1)

Where N is the total length of the sequence. Equation 1 cannot be applied straightforward to discriminate between correct and incorrect conformations because the magnitudes of each single term are very different: this is, the average value of some energy-terms (i.e. E_S3DC _and E_3DC_) have values around the standard deviation of others (i.e. E_local_, E_REF _and E_3D_). Consequently, we have defined a Zscore, named ZE (see equation 2). Zscores are obtained for each energy-term using a random distribution of residue-residue interactions per fold with the formulae: Zscore = (energy-*μ*)/*σ*, where "energy" is the energy-term calculated with the interactions of original sequence, *μ *is the average of this energy calculated with real and random interactions and *σ *its standard deviation. Random interactions between amino-acids are obtained by reshuffling the sequence of the protein. A total of 1000 random sequences are used to calculate the Zscore. The Zscore of an energy-term is identified with a Z prefix (i.e. Zscore of "x" energy-term is "Zx"). Hence, we calculate ZE_REF_, ZE_3D_, E_local_, ZE_S3DC _and ZE_3DC_. ZE_3D _is null because E_3D _is a constant value that depends only on the fold conformation. Also, the parameterization of E_REF _should not have any compensatory effect to discriminate between correct and incorrect folds. Therefore, we hypothesize that E_REF _should have similar distribution for real and random sequences and consequently ZE_REF _should fluctuate around 0. This also implies that the reference state function introduced in equation 1 by two energy terms, E_3D _and E_*REF*_, can be neglected by the use of Zscores (see results section for proofs).

We reformulate the Zscore in equation 2 with a linear combination and we define ZE by neglecting the term ZE_REF _and omitting the optimization of parameters (*α*_i_, with i = 1,2,3,4 in equation 2).(2)

To distinguish between terms calculated with statistical potentials obtained using the minimum distance (*min*-potential) or with C*β*-C*β *distances (C*β*-potential) we use the sub-index *min *and *Cβ*, respectively (i.e. for ZE we use ZE_min _and ).

In summary, we have two composite Zscores (ZE_min _and ) and six energy-terms (, , , ZE_*S3DC*-min_, ZE_*3DC*-min_, ZE_*local*-min_). ZE_*S3DC *_terms refer to the distance-dependent interaction between residues in specific local conditions. ZE_*3DC *_terms explain the distance-dependent interaction between local conditions, with independence of the residues involved. Finally, ZE_*local *_terms describe the cost to place one residue in a specific local condition. Because of the definitions of ZE_*3DC *_and ZE_*local *_they tend to positive values in folded structures. It is interesting to note that ZE_*local *_terms do not involve pairs of residues at certain distance, but only the requisites to accommodate a residue, buried or exposed, with a specific secondary structure.

## Results and discussion

### Development of an empirical scoring schema and parameter optimization

We first develop a new set of empirical potentials based on the theory formulated above. We split the database (1764 structural domains with non-homologous sequences from SCOP) in five groups and performed a 5-fold analysis of the data to extract the *φ *parameters required to calculate ZE_REF _and to check the distribution of the energy-terms of the potential (, , , ZE_*S3DC*-min_, ZE_*3DC*-min_, ZE_*local*-min_). A total of 209 *φ *parameters are obtained for pairs with local-conditions expressed as a triad of polar character, secondary structure and exposure degree with *min *and C*β *potentials. Although this amount of parameters might leave some doubts of a possible overfitting, we have to note that ZE_REF _is neglected on the evaluation of the scores for the prediction of correct folds (see equation 2), thus being irrelevant for the prediction and for the evaluation of the new scores.

**Figure 1:**
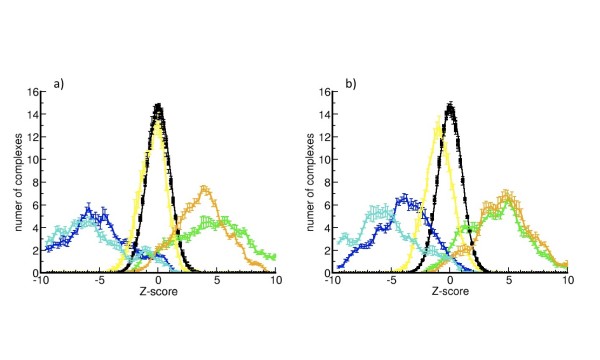
**5-fold average of the distribution of Zscores**. Average of the distribution of Zscores using a 5-fold approach plus the ranges of error. Zscores are calculated with *min*-potentials (a) and C*β*-potentials (b) for real conformations: ZE_S3DC _in blue, ZE_3DC _in green, ZE_local _in orange, ZE_REF _in yellow and ZE in cyan. In black are shown the distributions of the averages of all Zscores of randomly shuffled sequences and their error ranges.

The distributions of Zscores of the energy-terms of the potential are averaged using the results from the 5-fold validation procedure. Average distributions and standard errors of these Zscores calculated with C*β*-potentials and *min*-potentials are plotted in Figure 1. The comparison with the random set shows that the distribution of ZE_REF _of real conformations mostly overlaps with the distribution of randomly shuffled sequences using *min *or C*β*-C*β *force-fields. Consequently, we can neglect the contribution of *φ *parameters (yielding ZE_REF_) on the selection of the correct fold of a protein sequence, as stated previously and in the *Outline of the algorithm *section. ZE_local _and ZE_3DC _distributions accumulate positive scores (i.e. positive thresholds of both are required to identify near-native conformations). Interestingly, the deviation of ZE_local _with respect to the random distribution shows a low overlap, revealing the importance of the local conditions that apply on the protein sequence. This effect is the consequence that some residues are more comfortably accommodated on specific secondary structures, either exposed or buried, than others.

**Figure 2:**
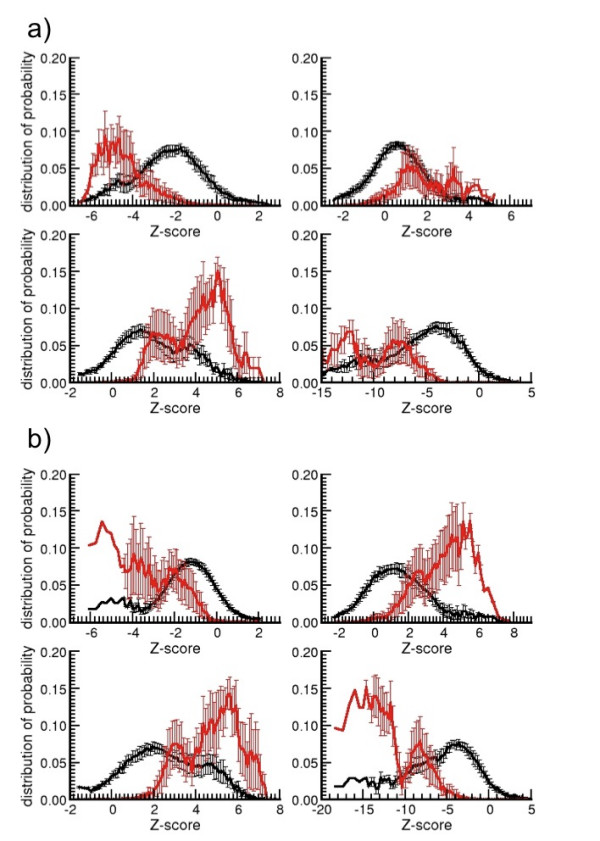
**Average of the distribution of probability of Zscores**. Average of the distribution of probability of Zscores (ZE_S3DC _in upper-left, ZE_3DC _in upper-right, ZE_local _in bottom-left and composite ZE in bottom-right) with *min*-potentials (a) and C*β*-potentials (b) in the set of MOULDER decoys. The distribution of probability is calculated as the ratio of the number of structures with a specific Zscore over the total. The distribution of probability for near-native structures is in red and the distribution of decoy models with non-native-like conformation is in black.

We construct the new potential with the total database of structures, formed by 1764 domains of SCOP with non-homologous sequences. Still, we need to prove the relevance and applicability of these new potentials. Therefore, the next step is to check if some of the energy-terms are more relevant than others to detect correctly folded structures or if the new composite scores (i.e. ZE_*min *_and ) require the information from each energy-term in equal proportion. This analysis is performed on a set of model-decoys derived from few target proteins with known structure. We used the set of decoys from MOULDER. This set contains several near-native structures (models which RMSD from its native structure is smaller than 3Å) from protein sequences that were not used on the generation of statistical potentials. We compare the Pearson product-correlation between the Zscores of energy-terms of the potential and the RMSD of the models for 20 target/model sets of decoys (Table 1). This shows a positive correlation between ZE_*min*_,  and the RMSD for many of the 20 target/model sets. Also, we compare the distribution of probability of scores of all energy-terms and composite Zscores of the model-decoys with the distribution of their near-native structures (figure 2.a for Zscores with *min *force-field and figure 2.b with C*β*-C*β *force-field). The distribution of probability is calculated as the ratio of the number of structures with a specific score over the total of decoys (for the distribution of scores of model-decoys) or the total of near-native structures (for the distribution of scores of near-native structures). The average of the distribution of the 20 sets of target/model decoys is shown in figures 2.a and 2.b. Because of averaging the distribution, some scores show a non-Gaussian behavior, presenting more than one maximum (in agreement with the correlations found among decoy sets in table 1). Positive values of ZE_*3DC *_and ZE_*local *_have higher occurrence in near-native structures than in non-native decoy models, while ZE_*S3DC *_of near-native structures are negative.

**Table 1:** Correlation between RMSD and Zscores in MOULDER.

Target					**ZE**_**min**_	**ZE**_***Aa3DEnv*-min**_	**ZE**_***3DEnv*-min**_	**ZE**_***local*-min**_
1bbh	0.86	0.36	-0.83	-0.80	0.62	0.42	-0.02	-0.71
1c2r	0.71	0.45	-0.48	-0.67	0.69	0.68	-0.43	-0.27
1cau	0.83	0.56	-0.71	-0.72	0.69	0.38	-0.40	-0.74
1cew	0.70	0.31	-0.64	-0.58	0.61	0.08	-0.18	-0.63
1cid	0.41	-0.12	-0.22	-0.59	0.45	0.43	0.10	-0.55
1dxt	0.87	0.75	-0.84	-0.76	0.78	0.76	-0.51	-0.51
1eaf	0.79	0.57	-0.61	-0.64	0.72	0.66	-0.38	-0.55
1gky	0.88	0.54	-0.77	-0.78	0.73	0.61	-0.18	-0.63
1lga	0.88	0.49	-0.68	-0.85	0.84	0.68	-0.40	-0.84
1mdc	0.78	0.50	-0.51	-0.64	0.68	0.40	-0.20	-0.60
1mup	0.85	0.66	-0.80	-0.83	0.80	0.79	0.24	-0.79
1onc	0.78	0.66	-0.58	-0.58	0.80	0.75	-0.29	-0.56
2afn	0.86	0.61	-0.60	-0.83	0.77	0.68	-0.27	-0.83
2cmd	0.81	0.68	-0.82	-0.78	0.63	0.58	-0.46	-0.65
2fbj	0.77	0.35	-0.32	-0.82	0.79	0.58	-0.13	-0.83
2mta	0.72	0.47	-0.16	-0.69	0.77	0.67	-0.02	-0.70
2pna	0.83	0.58	-0.79	-0.55	0.62	0.52	-0.29	-0.46
2sim	0.81	-0.12	-0.73	-0.81	0.66	0.20	-0.36	-0.76
4sbv	0.69	-0.10	-0.65	-0.60	0.54	0.46	-0.07	-0.45
8i1b	0.77	0.68	-0.36	-0.60	0.57	0.67	0.13	-0.43

Pearson product-correlation between Root Mean Square Deviation (RMSD) of MOULDER decoys of 20 target/model sets (in rows) and Zscores (in columns): , , , , ZE_min_, ZE_*S3DC*-min_, ZE_*3DC*-min_, and ZE_*local*-min_.

We also compare the *min *and C*β*-C*β *force-fields for the terms ZE_*S3DC*_, ZE_*3DC *_and ZE_*local*_. First, we observe that ZE_*3DC *_is a good descriptor to identify near-native structures when using the C*β*-C*β *force-field, but not with the *min *force-field. On the other hand, ZE_*S3DC *_is a good descriptor to identify near-native structures with the *min *force-field, but not with the C*β*-C*β *force-field. This indicates that the description of residues as hydrophobic or hydrophilic, their location in secondary structure and their degree of accessibility in the surface, are sufficient to identify the interacting pairs of a near-native fold when using a rough model of the backbone structure. Second, it is remarkable that the conditional location of residues produces a discriminative measure of the correct fold. This is related with the tendency of certain residues to be involved in specific secondary structures and with a particular degree of surface-accessibility. Besides, the definition of ZE_*local *_is virtually independent of the force-field used (*min *or C*β*-C*β*). Finally, both composite functions, ZE_min _and , take advantage of ZE_*local*_, while compensating ZE_*S3DC *_and ZE_*3DC *_into a single score. Still, we need to further compare them with other scoring functions in order to prove its utility to detect the native and near-native conformations among the sets of decoys.

### Detection of native conformations

To test the ability of the derived potentials to find the native conformation among different models we used four decoy data sets (*fisa_casp3, lmsd, 4state_reduced*, and MOULDER) and we compare ZE_*min *_and  with *DOPE, DFIRE, Prosa2003 *and *GA*_*341 *_(see methods and table 2). We find that most methods can successfully identify the native fold for over 15 targets. *DOPE *and *DFIRE *scores obtain best results in *fisa_casp3*, *lmsd*, and *4state_reduced *decoy sets, and ZE_*min *_is also successful. In summary, ZE_*min *_and  of the native conformations rank similar to *DOPE, DFIRE *and *Prosa2003 *in most targets. Thus, the utility of ZE_*min *_and  to detect the native conformation on a set of decoys is evinced and similar to *DOPE, DFIRE *and *Prosa2003*. Still, it would be interesting to explore further if ZE_*min *_and  help to find near-native conformations (not necessarily the native one) and to discard incorrect folds.

**Table 2:** Ranking position of the native structure according to the scoring functions.

Target set	DOPE	GA341	Prosa2003	DFIRE		ZEmin
fisa_casp3						

smd3	1	51	2	1	12	1
1bg8-A	1	808	151	1	341	2
1jwe	1	135	4	1	514	1
1eh2	8	826	93	1	577	159
1bl0	1	809	729	1	458	3

Total	4	0	0	5	0	2

lmds						

smd3	1	15	1	1	1	1
2ovo	7	33	1	61	115	7
1dtk	1	1	1	1	77	33
4pti	6	7	1	24	25	10
1b0n-B	293	35	1	418	99	180
1bba	501	395	458	501	389	63
1shf-A	1	5	13	1	199	1
1ctf	1	1	1	1	1	1
1fc2	501	234	107	501	276	489
1igd	1	1	1	18	10	1
2cro	1	10	1	1	43	16

Total	6	3	8	5	2	4

4state_reduced						

4rxn	1	1	8	1	20	26
4pti	1	6	1	1	1	1
1ctf	1	1	1	1	1	1
3icb	3	5	1	5	10	9
1sn3	1	1	1	1	25	3
2cro	1	5	1	1	1	2
1r69	1	4	1	1	1	1

Total	6	3	6	6	4	3

MOULDER						

1onc	1	1	1	1	1	1
1dxt	1	1	1	1	3	1
1eaf	1	1	1	1	1	1
1lga	1	1	1	1	1	1
1gky	1	1	1	1	1	1
1cau	1	1	1	1	1	1
4sbv	1	1	1	1	1	1
8i1b	1	1	1	1	1	1
2mta	1	1	1	1	4	4
2fbj	1	1	1	1	1	1
2cmd	1	1	1	1	1	1
1cew	1	1	1	1	1	1
2afn	1	1	1	1	1	1
2sim	1	1	1	1	1	1
1bbh	1	1	1	1	1	1
1mdc	1	1	1	1	1	1
1mup	1	1	1	1	15	1
2pna	85	45	43	85	58	9
1cid	1	1	1	1	1	1
1c2r	1	1	1	1	6	1

Total	19	19	19	19	15	18

Ranking position of the native structure among the sets of model/target decoys for several scoring functions. In the first column it is shown the code of the target protein used to generate the set of decoys. Next columns show the results for *DOPE, GA*_*341*_, *Prosa2003, DFIRE*, , ZE_*min *_scoring functions. The set of decoys is split in groups: MOULDER, *4state_reduced*, *fisa_casp3*, and *lmds*.

### Detection of near-native conformations

**Figure 3:**
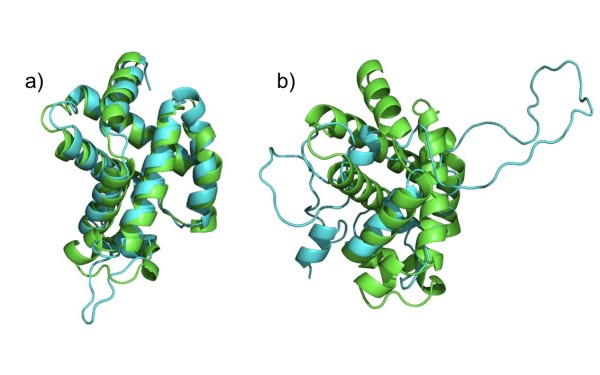
**Ribbon plot of **1dxt** native and decoy structures**. Ribbon plot of the native structure (in green) superposed with the model decoys (in cyan) of the target 1dxt in MOULDER. The structure of the decoy with smallest  score (model 51) is shown in 3.a and the structure of the decoy with highest  score (model 262) is shown in 3.b.

To test whether the derived potentials are able to identify near-native conformations among the set of decoy structures, we define the nearest-native conformation of a target as the model with the smallest RMSD to the target native conformation different than zero. In a similar design as for table 2, we calculate the RMSD difference (ΔRMSD) between the RMSD of the best non-native candidate and the RMSD of the nearest-native conformation (see table 3) [12,31,38]. The best candidates are chosen using the scores of *DOPE, DFIRE, Prosa2003, GA*_*341*_, ZE_*min *_and  among the set of models excluding the native conformation. Figure 3 shows the superposition of the native structure with the best and the worst candidates from the decoys of target "1dxt" in MOULDER. As expected, ΔRMSDs are large for most models of *fisa_casp3 *and *lmsd *decoys and small on sets of *4state_reduced *and MOULDER. The smallest values of the average of ΔRMSD are obtained with *DFIRE*, ZE_*min *_and  in MOULDER model/target sets while for the *4state_reduced *set the smallest averaged ΔRMSDs are obtained with *Prosa2003 *and ZE_*min*_. However, it has to be noted that ZE_*min *_uses information of side-chain conformation, while classical functions *Prosa2003, DFIRE, DOPE *and *GA*_*341 *_use only information of C*β *atoms.

**Table 3:** ΔRMSD according to several scoring functions on the set of model/target decoys.

Target set	DOPE	GA341	Prosa2003	DFIRE		ZEmin
**fisa_casp3**						

1eh2	6,06	4,64	1,64	4,93	4,13	4,93
1bg8-A	7,84	7,28	7,28	3,58	5,72	3,58
1jwe	6,30	10,62	9,75	8,10	9,52	8,10
1bl0	4,10	2,24	2,24	7,10	4,45	7,10
smd3	4,35	6,44	5,08	5,12	5,32	4,47

Average	5,73	6,25	5,20	5,76	5,83	5,63

lmds						

1dtk	5,46	4,75	4,59	4,59	4,89	2,90
1igd	7,64	1,63	4,28	5,61	4,50	5,61
2cro	8,68	8,95	6,14	10,01	5,93	9,48
smd3	4,35	4,52	2,68	5,52	2,50	5,52
1ctf	9,41	7,65	7,52	7,37	6,67	7,37
1fc2	0,26	0,51	1,00	0,07	1,51	0,07
1shf-A	5,83	5,16	3,06	6,91	5,24	6,91
4pti	5,64	5,72	9,91	4,61	9,54	4,61
2ovo	6,92	3,49	6,45	5,70	7,26	5,70
1b0n-B	1,60	2,20	0,61	0,50	1,76	0,50
1bba	1,89	0,87	0,59	3,29	2,00	1,92

Average	5,24	4,13	4,26	4,92	4,71	4,60

4state_reduced						

1sn3	1,69	0,90	4,09	4,71	6,05	0,90
1r69	2,55	0,80	0,79	0,95	2,29	0,79
4pti	0,82	5,53	0,07	0,07	1,18	2,80
2cro	2,46	1,24	0,29	1,24	0,53	0,53
1ctf	0,33	0,60	0,50	2,93	1,02	1,02
3icb	1,86	1,51	0,93	0,11	0,05	0,11
4rxn	0,46	3,52	0,75	0,70	0,68	0,70

Average	1,45	2,01	1,06	1,53	1,69	0,98

MOULDER						

1onc	1,16	0,72	0,60	0,40	0,40	0,40
1dxt	3,97	0,00	0,55	1,11	0,00	0,55
1eaf	0,34	1,72	1,72	0,47	0,99	0,47
1lga	0,82	5,89	5,89	0,80	0,00	0,80
1gky	0,57	0,34	0,57	0,57	0,62	0,57
1cau	3,89	1,95	0,42	0,42	0,07	0,42
4sbv	0,00	5,57	0,00	0,00	6,43	0,00
8i1b	0,38	0,42	0,39	0,50	0,36	1,04
2mta	0,31	0,57	0,21	0,63	0,32	0,63
2cmd	0,38	2,22	0,58	0,23	0,74	0,84
2fbj	0,26	2,80	0,32	0,91	0,51	0,91
1cew	2,06	2,73	2,73	3,47	3,73	3,47
2afn	0,71	0,75	0,68	0,12	0,50	0,12
2sim	1,21	0,42	0,46	0,16	1,13	0,16
1bbh	0,88	0,11	0,16	0,00	0,31	0,00
1mdc	0,03	0,74	6,85	0,16	0,00	0,16
1mup	0,53	0,17	0,67	0,67	0,32	0,46
2pna	0,26	0,60	0,42	0,24	0,26	0,24
1cid	1,15	1,15	1,15	0,08	1,15	1,15
1c2r	3,42	0,00	0,85	0,00	0,00	0,15

Average	1,12	1,44	1,26	0,55	0,89	0,63

In the first column it is shown the code of the target protein used to generate the set of decoys. Next columns show the ΔRMSD for *DOPE, GA*_*341*_, *Prosa2003, DFIRE*, , ZE_*min *_scoring functions. The set of decoys is split in groups: MOULDER, *4state_reduced*, *fisa_casp3*, and *lmds*.

**Figure 4:**
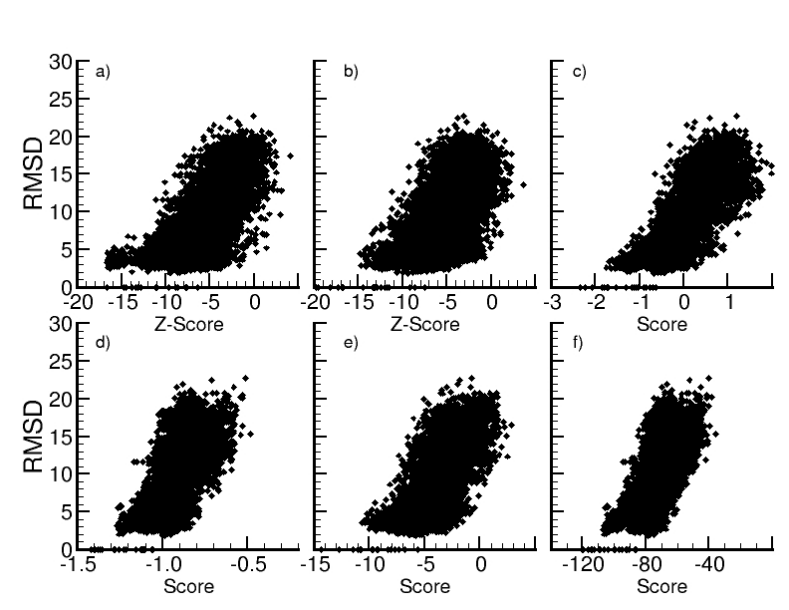
**Comparison of RMSD/score resulting from several scoring functions**. Root Mean Square Deviations (RMSD) of MOULDER decoys are plotted versus Zscores of  (a) and ZE_min _(b), and versus scores normalized by the length of the sequence of *Prosa2003 *(c), *DFIRE *(d), *GA*_*341 *_(e), and *DOPE *(f).

**Figure 5:**
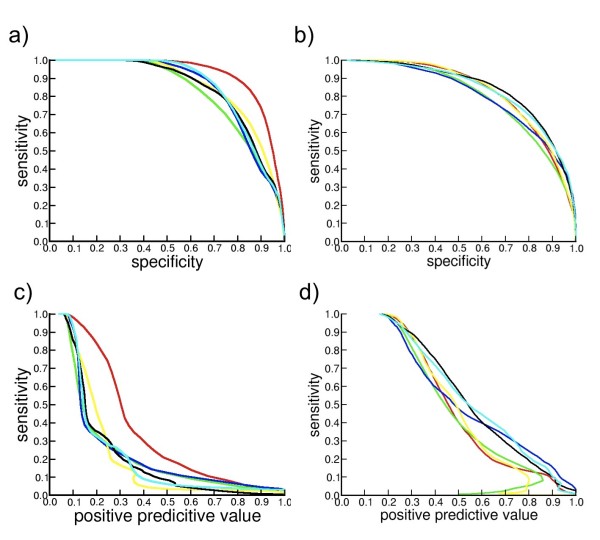
**ROC curves of scoring functions applied in MOULDER and *4state_reduced *sets**. Sensitivity is plotted versus specificity and Positive Predictive Value (PPV) for all decoy conformations from MOULDER set (5.a and 5.c) and from *4state_reduced *set (5.b and 5.d). Scoring functions used are: *Prosa2003 *(red), *DFIRE *(green), *DOPE *(blue), *GA*_*341 *_(yellow),  (black) and ZE_*min *_(cyan).

We use the same MOULDER decoy set to compare the RMSD and the scores calculated with , ZE_min_, *DOPE, DFIRE, GA*_*341 *_and *Prosa2003 *(Figure 4). ROC curves of sensitivity/specificity and sensitivity/PPV are calculated with all conformations from the sets of models from MOULDER and *4state_reduced *(Figure 5). They show the ability of  and ZE_min _to identify wrong conformations without lost of coverage but less capacity to detect near-native conformations. We use the program StaR [60] to assess the statistical significance of the observed difference between these scoring functions when used as binary classifiers (see Additional files 2 and 3: Supplemental tables S2 and S3). With the set of MOULDER decoys (figures 5.a and 5.c) the scoring functions , ZE_min_, *DOPE *and *GA*_*341 *_show similar performance if we consider that for p-values smaller than 0.05 the difference is significant. With the set of *4state_reduced *decoys (figures 5.b and 5.d) only the difference between  and *GA*_*341 *_have significant p-value higher than 0.0005 and we can assume that the differences among all scoring functions are significant.

**Figure 6:**
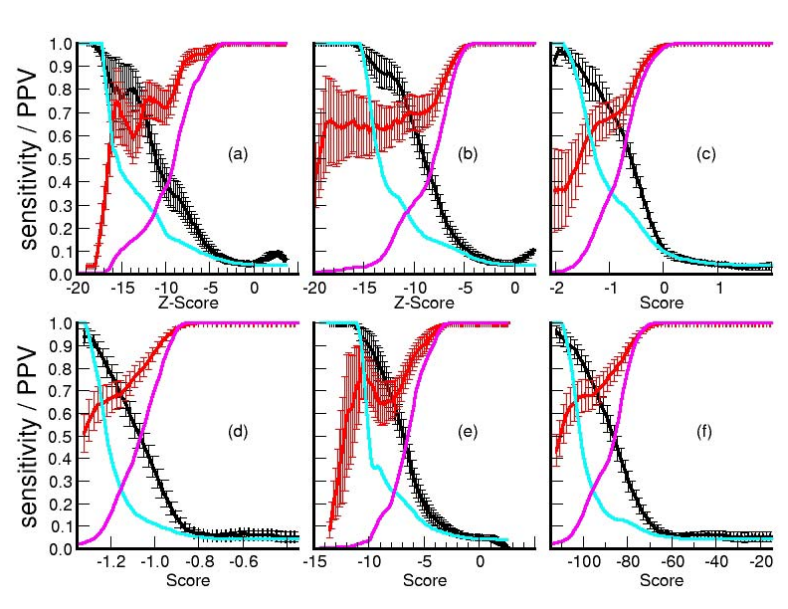
**Sensitivity and PPV versus scoring functions applied in MOULDER decoy set**. Average and standard error of sensitivity (red) and PPV (black) are calculated with the predictions in 20 target/model groups and total sensitivity (purple) and PPV (cyan) with the total of decoy models in MOULDER set. Score functions are:  (a), ZE_min _(b), *Prosa2003 *(c), *DFIRE *(d), *GA*_*341 *_(e), and *DOPE *(f).

**Figure 7:**
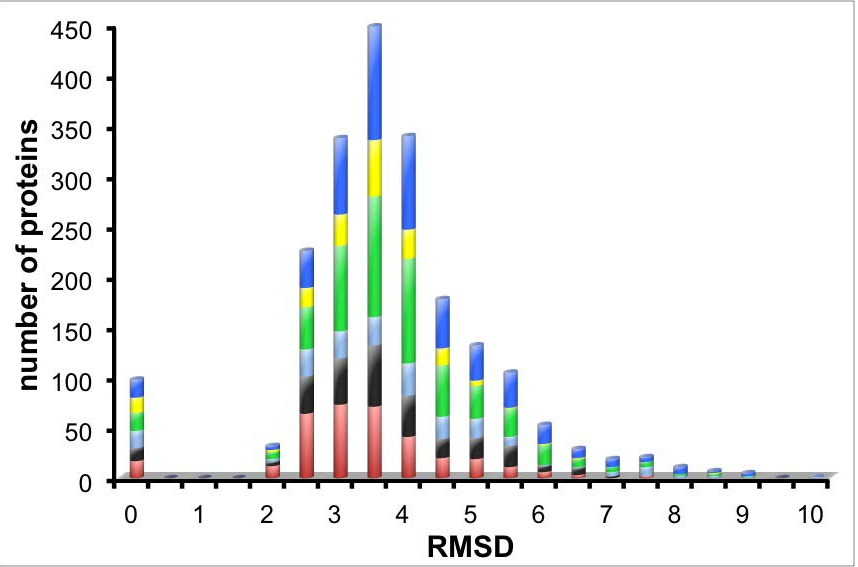
**Distribution of RMSD of decoy-models in MOULDER set**. Decoy structures predicted as positive for each scoring function are compared with their targets. The plot accumulates the predictions of the scoring methods:  (black), ZE_min _(cyan), *Prosa2003 *(red), *DFIRE *(green), *GA*_*341 *_(yellow), and *DOPE *(blue). Most positives are found within less than 5Å from the original structure.

PPV and sensitivity curves with respect to scores and Zscores are used to select a threshold to accept a putative conformation. Figure 6 shows the plot of the average (plus error deviations) of PPV and sensitivity of the 20 model/target sets on MOULDER decoys versus the thresholds used. Also the total PPV and sensitivity is calculated with all models and plotted in Figure 6. The Zscore (or score) at the cross points between the curves with the total PPV and sensitivity produce high values of average PPV and sensitivity for all methods. These cross-points obtain a good balance between total PPV and sensitivity for each method. Therefore, conformations with Zscores lower than their thresholds were accepted as correct predictions (positives). The distribution of RMSDs among positives of the scoring-functions indicates that  works as many other methods (in agreement with the significances calculated with StaR). Also, most positives have RMSD smaller than 5Å (Figure 7). More than 50% of true positives in MOULDER set were obtained either with *Prosa2003 *(occasionally by some other method besides *Prosa2003*) or by all methods except *Prosa2003 *(*DFIRE, DOPE, GA*_*341*_,  and ZE_min_). The remaining set of true-positives is obtained by many scoring functions and often by more than one (tables 4 and 5). Interestingly, all scoring functions discriminate well among the set of true-negatives (wrong conformations) in MOULDER. Moreover, almost 50% of false positives are found among those conformations accepted by *DOPE*, *DFIRE *and *Prosa2003*. The use of  ensures a large amount of conformers which structure differed from the native conformation by less than 3.5Å, while purging more than 80% of spurious conformations. Therefore,  and ZE_min _are not redundant with any of the classical scoring functions, while in combination with them they may help to cover a larger set of correct conformations.

**Table 4:** Statistical analysis of positives by scoring functions in MOULDER set.

*True Positives*		*False Positives*	
***Combination of scores***	***#decoys***	***Combination of scores***	***#decoys***

*Prosa2003*	34	*DFIRE*; *DOPE*	126
; ZE_min_; *DFIRE*; *GA*_*341*_; *DOPE*	23	*Prosa2003*; *DFIRE*; *DOPE*	81
*Prosa2003*; ; *DFIRE*; *DOPE*;	19	*Prosa2003*	72
*Prosa2003*; ; ZE_min_; *DFIRE*; *GA*_*341*_; *DOPE*	14	ZE_min_	48
*Prosa2003*; *DFIRE*; *DOPE*	12	*Prosa2003*; ; *DFIRE*; *GA*_*341*_; *DOPE*	35
*Prosa2003*; *DFIRE*	10	; ZE_min_; *DFIRE*; *GA*_*341*_; *DOPE*	34
ZE_min_	9	*DFIRE*	33
*Prosa2003*;	8	; *DFIRE*; *DOPE*	31
*DFIRE*; *DOPE*	3	*DOPE*	31
*Prosa2003*; ZE_min_	2		24
; ZE_min_; *DFIRE*; *DOPE*	2	; *DFIRE*; *GA*_*341*_; *DOPE*	18
*Prosa2003*; ZE_min_; *DFIRE*; *GA*_*341*_; *DOPE*	2	ZE_min_; *DFIRE*; *DOPE*	17
ZE_min_; *GA*_*341*_	2	*Prosa2003*; ; *DFIRE*; *DOPE*	16
*Prosa2003*; ZE_min_; *GA*_*341*_	1	; ZE_min_; *DFIRE*; *DOPE*	15
ZE_min_; *DFIRE*; *DOPE*	1	*GA*_*341*_	13
	1	*Prosa2003*; *DFIRE*; *GA*_*341*_; *DOPE*	13
; *DFIRE*; *GA*_*341*_; *DOPE*	1	; ZE_min_	10
; ZE_min_; *GA*_*341*_	1	ZE_min_; *DFIRE*	7
*Prosa2003*; ; *DOPE*	1	*Prosa2003*;	6
ZE_min_; *DFIRE*; *GA*_*341*_; *DOPE*	1	*DFIRE*; *GA*_*341*_; *DOPE*	5
*GA*_*341*_	1	*Prosa2003*; ; *GA*_*341*_	3
*Prosa2003*; ; ZE_min_; *DFIRE*; *DOPE*	1	; ZE_min_; *DFIRE*	3
; *DFIRE*; *DOPE*	1	*Prosa2003*; ; ZE_min_; *DFIRE*; *GA*_*341*_; *DOPE*	3
		*Prosa2003*; *DOPE*	3
		ZE_min_; *DOPE*	3
		*Prosa2003*; *DFIRE*;	3
		; ZE_min_; *DFIRE*; *GA*_*341*_	3
		; *DOPE*	3
		ZE_min_; *GA*_*341*_	3
		; *GA*_*341*_	1
		; *DFIRE*	1
		; ZE_min_; *GA*_*341*_	1

Distribution of true-positives and false-positives among decoys of MOULDER according to one or more scoring functions and their thresholds. Columns show the number of decoys (*#decoys*) found by one or more scoring functions (*combination of scores*).

**Table 5:** Statistical analysis of negatives by scoring functions in MOULDER set.

*True Negatives*		*False Negatives*	
***Combination of scores***	***#decoys***	***Combination of scores***	***#decoys***

*Prosa2003*; ; ZE_min_; *DFIRE*; *GA*_*341*_; *DOPE*	4708	*Prosa2003*; ; ZE_min_; *DFIRE*; *GA*_*341*_; *DOPE*	81
*Prosa2003*; ; ZE_min_; *GA*_*341*_	126	; ZE_min_; *DFIRE*; *GA*_*341*_; *DOPE*	34
; ZE_min_; *GA*_*341*_	81	*Prosa2003*	23
; ZE_min_; *DFIRE*; *GA*_*341*_; *DOPE*	72	ZE_min_; *GA*_*341*_	19
*Prosa2003*; ; *DFIRE*; *GA*_*341*_; *DOPE*	48	; ZE_min_; *GA*_*341*_	12
*Prosa2003*; *DFIRE*; *GA*_*341*_; *DOPE*	46	; ZE_min_; *GA*_*341*_; *DOPE*	10
ZE_min_	35	*Prosa2003*; ; *DFIRE*; *GA*_*341*_; *DOPE*	9
*Prosa2003*	34	ZE_min_; *DFIRE*; *GA*_*341*_; *DOPE*	8
*Prosa2003*; ; ZE_min_; *GA*_*341*_; *DOPE*	33	*Prosa2003*; ; ZE_min_; *GA*_*341*_	3
*Prosa2003*; ; ZE_min_; *DFIRE*; *GA*_*341*_	31		2
*Prosa2003*; ZE_min_; *GA*_*341*_	31	; *DFIRE*; *GA*_*341*_; *DOPE*	2
*Prosa2003*; ZE_min_; *DFIRE*; *GA*_*341*_; *DOPE*	24	*Prosa2003*; *GA*_*341*_	2
*Prosa2003*; ZE_min_	18	*Prosa2003*; ; *DFIRE*; *DOPE*	2
*Prosa2003*; ; *GA*_*341*_	17	*Prosa2003*; ZE_min_; *GA*_*341*_	1
ZE_min_; *GA*_*341*_	16	*Prosa2003*; ; *GA*_*341*_	1
*Prosa2003*; *GA*_*341*_	15	*Prosa2003*; *DFIRE*; *DOPE*	1
; ZE_min_	13	*Prosa2003*; ZE_min_	1
*Prosa2003*; ; ZE_min_; *DFIRE*; *DOPE*	13	*Prosa2003*; ZE_min_; *DFIRE*; *GA*_*341*_; *DOPE*	1
*Prosa2003*; ; *GA*_*341*_; *DOPE*	7	*GA*_*341*_	1
ZE_min_; *DFIRE*; *GA*_*341*_; *DOPE*	6	*Prosa2003*; ; ZE_min_; *DFIRE*; *DOPE*	1
*Prosa2003*; ; ZE_min_	5	ZE_min_; *DFIRE*; *GA*_*341*_	1
ZE_min_; *DFIRE*; *DOPE*	3	; *DFIRE*; *DOPE*	1
*Prosa2003*; ; *DFIRE*; *GA*_*341*_	3	*Prosa2003*;	1
; ZE_min_; *GA*_*341*_; *DOPE*	3		
*Prosa2003*; *DOPE*	3		
; ZE_min_; *DFIRE*; *GA*_*341*_	3		
*Prosa2003*; ZE_min_; *DFIRE*; *GA*_*341*_	3		
*Prosa2003*; ; *DFIRE*; *DOPE*	3		
*Prosa2003*; *GA*_*341*_; *DOPE*	3		
*Prosa2003*; *DFIRE*; *DOPE*	1		
*Prosa2003*; ZE_min_; *GA*_*341*_; *DOPE*	1		
*Prosa2003*; ZE_min_; *DFIRE*; *DOPE*	1		

Distribution of true-negatives and false-negatives among decoys of MOULDER according to one or more scoring functions and their thresholds. Columns show the number of decoys (*#decoys*) found by one or more scoring functions (*combination of scores*).

In summary, the utility of  to detect near-native structures has been attested. Moreover, the global-statistic results (PPV, sensitivity, RMSD distribution, etc.) are similar to state-of-the-art methods like *DOPE, DFIRE, GA*_*341 *_and *Prosa2003*, but the individual results for each particular decoy conformer are different. This proves the convenience of using  in combination with other methods. More in detail, most near-native conformations are found by more than 50% of methods, but few of them are detected by one or at most two methods. Thus, it is convenient to use more than one method to confirm a prediction and to increase the coverage of near-native structures. Even though the best results are obtained with *Prosa2003*, the combination with *DFIRE, DOPE, GA*_*341*_,  and ZE_min _can increase the coverage up to 50%, while the number of non-native-like conformations is not largely distended. The best strategy to detect near-native structures is to use a composite score (i.e. *QMEAN*[12] or a SVM composite score[38]). Here we have proved that: 1)  and ZE_min _can detect near-native structures undetected by other methods, thus it is worth to use them with other composite scores; 2)  and ZE_min _are already composite functions that can itself be improved using weights for each individual component; and 3) each component term of  and ZE_min _disclose the features of residue-pair interactions and the local environment of residues, thus they can be used to detect the main components affecting the structure either to be considered near-native (stabilizing) or non-native-like (destabilizing). Still, besides characterizing the main components affecting the Zscore it is usually interesting to identify the region of the structure stabilizing or destabilizing the protein conformation, not only the energetic component affected (i.e. residues with wrong secondary structure assignment or with unfeasible interactions). This implies to distribute the Zscore along the sequence. However, only those methods scoring the energy in a sum of terms per residue can split the score along the protein sequence. This is possible only for few methods (e.g. *Prosa2003 *or *DOPE*), but not for all and even more difficult for composite functions. The use of Zscores instead of original energies (i.e. , , , E_*S3DC*-min_, E_*3DC*-min_, and E_*local*-min_) impedes its distribution along the protein sequence because by definition it cannot produce a sum of terms per residue. In the next section is presented an approach to distribute the Zscore of a model structure along its protein sequence and its applicability to detect local errors in the structure.

### Detection of local errors in the conformation of decoy models

**Figure 8:**
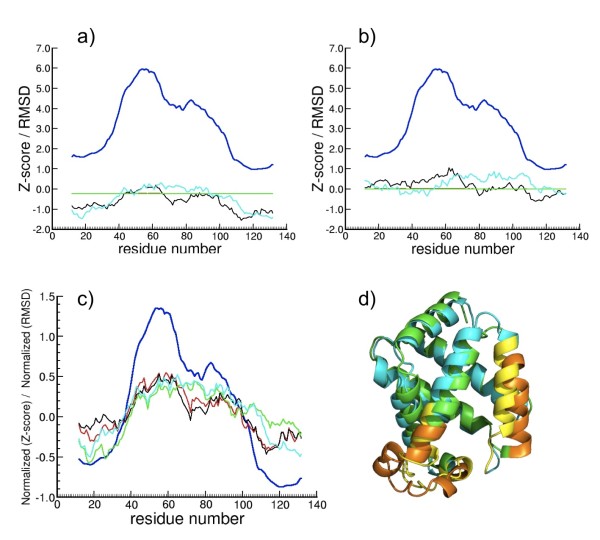
**Comparison of RMSD and residue-position Zscores for target **1dxt** in MOULDER**. Comparison of RMSD of the C*α *trace of a decoy conformer (model 113) of target 1dxt in MOULDER and its residue-position Zscores Sc, ScZE_min_,  and Z_A_E_min_. 8.a) RMSD is compared with residue-position Zscores. 9.b) RMSD is compared with the difference of residue-position Zscores between the model and the native structure (1dxt). 8.c) Residue-position Zscores and RMSD values of the C*α *trace are normalized along the sequence and compared. Feature colors are: RMSD in blue, Sc in red, ScZE_min _in green,  in black and Z_A_E_min _in cyan. 8.d) The native structure of 1dxt is shown in ribbons (green) superposed with the structure of the near-native decoy (model 113, in cyan), showing the fragments with higher residue-position Zscores and RMSD in orange (native) and yellow (model 113).

The RMSD between C*α *atoms of the decoy-model conformations in MOULDER and their corresponding target are compared to Sc, ScZE_min_,  and Z_A_E_min _(see methods). On the one hand we compare the RMSD and the residue-position Zscores of the models. We expect that the highest RMSD between C*α *atoms (i.e. in regions wrongly modeled) will have the highest scores (see example in Figure 8.a). On the other hand, we compare the C*α *RMSDs' with the difference of residue-position Zscores between each decoy-model and its target (see example in Figure 8.b). Due to the different magnitudes of RMSDs and Zscores, these curves have to be normalized for the sake of comparison. The normalized values are defined as (X_i _- <X>)/*σ *where X_i _is either any of the Zscores on position *i *or the C*α *RMSD of residue *i*, <X> is the average along the sequence and *σ *the standard deviation (see Figure 8.c). The coincidence of picks in RMSD and Zscore curves identifies the differences detected between the near-native and decoy structures (Figure 8.d).

The Pearson product-correlation between the C*α *RMSDs' and the residue-position Zscores of the model decoys (or its difference with respect to their targets) show the possibilities to use the Zscores to detect the accuracy of the models (see Table 6). In general, residue-position Zscores of decoy structures work better than Zscore differences with respect to the original target to validate local conformation, and Zscores based on C*β*-potentials are better than *min*-potentials. Nonetheless, the number of times that the Pearson correlation is higher than 0.5 for models with backbone RMSD smaller than 7Å with respect to the target is not large enough to guarantee its use for identifying locally erroneous conformations. Potentials (and Zscores) of a residue or a continuous fragment of residues are affected by the rest of the protein-sequence. Therefore, regions with near-native conformation may have peaks of energy (and Zscore) due to other regions wrongly modeled. This diminishes the correlation between C*α *RMSDs and local residue-position scores. Interestingly, there is a remarkable correlation between Sc and  and between ScZE_min _and Z_A_E_min _(e.g. in figure 8.c): 1881 out of 2107 models with RMSD smaller than 7Å have Pearson correlation higher than 0.5 between Sc and  (averaging about 0.82 ± 0.15), while 1778 out of 2107 had Pearson correlation between ScZE_min _and Z_A_E_min _higher than 0.5 (averaging about 0.77 ± 0.15). This supports the use of just one of the methods for the assessment of the local conformation.

**Table 6:** Correlation between RMSD and residue-position Zscores

Target Set									C(ΔZ_**A**_E_***min***_)		C_**L**_(Z_**A**_E_***min***_)		C(ΔScZE_***min***_)		C_**L**_(ScZE_***min***_)	
	
	Average	P/N	Average	P/N	Average	P/N	Average	P/N	Average	P/N	Average	P/N	Average	P/N	Average	P/N
1bbh	0.7 ± 0.1	124/34	0.7 ± 0.1	82/76	0.7 ± 0.1	135/23	0.7 ± 0.1	109/49	0.7 ± 0.1	112/46	0.6 ± 0.1	37/121	0.6 ± 0.1	92/66	0.7 ± 0.1	56/102
1c2r	0.7 ± 0.1	87/22	0.6 ± 0.2	25/84	0.7 ± 0.1	62/47	0.6 ± 0.2	23/36	0.5 ± 0.2	10/99	0.6 ± 0.2	13/96	0.5 ± 0.2	6/103	0.5 ± 0.2	5/104
1cau	0.6 ± 0.2	22/16	0.7 ± 0.2	28/10	0.5 ± 0.2	4/34	0.7 ± 0.2	28/10	0.6 ± 0.2	11/27	0.7 ± 0.2	26/12	0.5 ± 0.2	7/31	0.6 ± 0.2	19/19
1cew	0.8 ± 0.0	1/11	0.5 ± 0.3	3/9	0.6 ± 0.0	1/11	0.8 ± 0.0	1/11	0.6 ± 0	1/11	0.5 ± 0.4	2/10	0.0 ± 0.0	0/12	0.8 ± 0.0	1/11
1cid	0.7 ± 0.1	56/27	0.8 ± 0.2	52/31	0.7 ± 0.1	60/23	0.7 ± 0.1	59/24	0.8 ± 0.1	77/6	0.7 ± 0.2	28/55	0.8 ± 0.1	80/3	0.7 ± 0.2	28/55
1dxt	0.6 ± 0.1	44/106	0.7 ± 0.1	89/61	0.6 ± 0.1	79/71	0.7 ± 0.1	111/39	0.5 ± 0.2	6/144	0.7 ± 0.2	46/104	0.5 ± 0.2	8/142	0.7 ± 0.1	56/94
1eaf	0.5 ± 0.2	10/50	0.6 ± 0.2	18/42	0.6 ± 0.2	15/45	0.5 ± 0.1	22/38	0.4 ± 0.2	3/57	0.6 ± 0.2	17/43	0.4 ± 0.2	3/57	0.6 ± 0.1	28/32
1gky	0.6 ± 0.2	12/17	0.7 ± 0.2	14/5	0.5 ± 0.2	5/14	0.6 ± 0.2	14/5	0.6 ± 0.2	13/6	0.6 ± 0.2	10/9	0.6 ± 0.2	13/6	0.7 ± 0.2	12/7
1lga	0.5 ± 0.2	12/95	0.5 ± 0.2	10/97	0.5 ± 0.2	9/98	0.5 ± 0.2	5/102	0.6 ± 0.0	1/106	0.5 ± 0.2	8/99	0.4 ± 0.3	2/105	0.4 ± 0.3	3/104
1mdc	0.7 ± 0.1	59/56	0.7 ± 0.2	42/73	0.7 ± 0.1	68/47	0.7 ± 0.2	47/68	0.6 ± 0.1	39/76	0.6 ± 0.1	28/87	0.6 ± 0.2	16/99	0.6 ± 0.2	18/97
1mup	0.7 ± 0.1	60/74	0.7 ± 0.1	73/61	0.7 ± 0.1	68/66	0.7 ± 0.1	77/57	0.7 ± 0.1	99/35	0.7 ± 0.1	59/75	0.7 ± 0.1	112/22	0.7 ± 0.1	53/81
1onc	0.7 ± 0.2	53/69	0.6 ± 0.2	29/93	0.7 ± 0.2	59/63	0.7 ± 0.2	38/84	0.7 ± 0.1	102/20	0.7 ± 0.2	43/79	0.7 ± 0.1	86/36	0.7 ± 0.1	40/82
2afn	0.6 ± 0.1	80/39	0.6 ± 0.1	73/46	0.6 ± 0.1	22/97	0.6 ± 0.1	71/48	0.5 ± 0.1	25/94	0.5 ± 0.2	18/103	0.5 ± 0.2	9/110	0.5 ± 0.1	17/102
2cmd	0.7 ± 0.1	101/128	0.6 ± 0.1	112/117	0.6 ± 0.1	93/136	0.6 ± 0.1	103/126	0.6 ± 0.1	102/127	0.6 ± 0.1	50/179	0.6 ± 0.1	42/187	0.6 ± 0.1	58/171
2fbj	0.6 ± 0.2	12/89	0.6 ± 0.1	24/77	0.6 ± 0.2	7/94	0.6 ± 0.2	13/88	0.6 ± 0.1	32/69	0.6 ± 0.2	11/90	0.6 ± 0.1	40/61	0.6 ± 0.1	19/82
2mta	0.6 ± 0.1	47/111	0.7 ± 0.1	48/110	0.7 ± 0.1	75/83	0.7 ± 0.2	32/126	0.7 ± 0.1	104/54	0.7 ± 0.1	73/85	0.7 ± 0.1	130/28	0.6 ± 0.2	40/118
2pna	0.7 ± 0.1	41/97	0.7 ± 0.1	73/65	0.7 ± 0.2	37/101	0.7 ± 0.1	71/67	0.0 ± 0.0	0/138	0.7 ± 0.2	67/71	0.6 ± 0.2	12/126	0.7 ± 0.2	59/79
2sim	0.5 ± 0.2	4/90	0.4 ± 0.3	3/91	0.0 ± 0.0	0/94	0.6 ± 0.0	1/93	0.4 ± 0.3	2/92	0.6 ± 0.0	1/93	0.4 ± 0.3	2/92	0.0 ± 0.0	0/94
4sbv	0.5 ± 0.4	2/2	0.4 ± 0.3	2/2	0.4 ± 0.3	2/2	0.4 ± 0.3	2/2	0.0 ± 0.0	0/4	0.0 ± 0.0	0/4	0.0 ± 0.0	0/4	0.0 ± 0.0	0/4
8i1b	0.4 ± 0.3	2/135	0.6 ± 0.2	11/126	0.5 ± 0.2	5/132	0.6 ± 0.2	13/124	0.6 ± 0.1	43/94	0.5 ± 0.1	18/119	0.5 ± 0.1	23/114	0.6 ± 0.2	13/124

Pearson correlation between RMSD of C*α *atoms and residue-position Zscores of structure-models in MOULDER decoy set. In the first column is shown the code of the native protein used to generate the decoys of a model/target set. Next columns show: i) the average of Pearson correlation (*Average*) of those models with RMSD from the native structure smaller than 7Å and using only correlations higher than 0.5; and ii) the ratio P/N, being P the number of models with correlation larger than 0.5 and N those with correlation smaller than or equal to 0.5 among models with RMSD larger than 7Å. Residue-position Zscores are: Sc, ScZE_min_, , Z_A_E_min _and the differences of Sc and ScZE_min _of the decoy conformers with respect to their native structure (ΔSc and ΔScZE_min_). Pearson correlations between C*α *RMSDs and Zscores are denoted as C(Zscore) - in even columns -, while correlation of C*α *RMSDs and Zscores normalized by length are indicated as C_L_(Zscore) - in odd columns -.

In summary, we have introduced the equations to distribute the protein Zscore along its sequence. We have also provided some evidence of their utility to identify regions where the conformation deviates from the native structure. However, further analyses are needed to fully prove the use of the local Zscores, by remodeling local fragments of the structure and recalculating the Zscores, but this is beyond the scope of the present work.

## Conclusion

We have introduced a method to split knowledge-based potentials and to solve the definition of the reference state. We have defined two scoring functions as linear combinations of energetic terms, transformed into a sum of Zscores and proved that the functions containing the reference state could be neglected on both. There is room still for improvement using machine-learning approaches or optimization rules, like support vector machines or artificial neural networks, to assign the weights of the linear combination of energy-terms. With the simplest approach we obtained predictions similar to the state-of-the-art of other methods (i.e. *Prosa2003*, *DOPE*, *GA*_*341*_, or *DFIRE*) for several testing decoy sets. This included finding the native conformation or finding the closest set of conformers to the native structure (i.e. RMSD smaller than 3Å). It is remarkable that some predictions were not obtained by some classical approaches (i.e. *Prosa2003*, *DOPE *or *DFIRE*) but were obtained using .

Finally, we defined four scoring approaches for local conformation in order to find errors on model structures. We found a good correlation between the residue-position Zscore (i.e.  and Z_A_E_min_) and the residue-scanning Zscore (i.e. Sc and ScZE_min_), which allow us to use the less expensive computational approach (residue-position Zscore) to analyze the local conformation. We compared the residue-position Zscores with the local RMSD of C*α *atoms and proved that it can be used to identify wrongly modeled regions.

## Methods

### Development of statistical potentials

We developed the statistical potentials used in this study from an independent dataset of 1764 structural domains extracted from SCOP[61]. These domains corresponded to non-homologous sequences (with less than 40% sequence similarity). Splitting the data in five equivalent groups performed the 5-fold validation procedure. Frequency-contacts, statistical potentials and Zscores of the energy-terms were calculated with four of them and the Zscores of the remaining set were compared with random distributions of their sequences (dividing the results of the randomly shuffled sequences by 1000 in order to visualize a 1/1 ratio for all distributions). The procedure was repeated five times (5-fold) for the shake of robustness of the results. Also the values of *φ *were obtained five times by fitting the scores and its deviations were compared (see Additional file 4: supplemental table S1).

### Database of decoy structures

We have used decoy structures to test and compare several scoring functions in order to reveal which one is the best at identifying near-native conformations. Several sets of decoys are used that include structures close to the native X-ray structure and show native-like properties of the real folded conformation[62]. Besides, these sets contain numerous models showing many different arrangements for statistical analysis purposes. Two main decoy databases were used to test ZE scores: i) MOULDER decoy set[63] contains 300 models from 20 target/template pairs sharing low sequence identity (i.e. each of the models for a given target were of the same sequence and length); and ii) Decoys'R'Us database[64] contains a variety of decoys generated by different methods with the aim of fooling scoring functions. We have used three sets from the second database of decoys: *4state_reduced *(around 600 models for 7 target proteins[65]) contains several native-like conformations built using a 4-state off-lattice model, while most decoys in *lmds *(around 400 models for 11 target proteins[50]) and *fisa_casp3 *(around 1400 models for 5 target proteins[55]) have models with large RMSD with respect to the native conformation. Consequently, these sets show different properties for the analysis: MOULDER decoy set and *4state_reduced *set are used to test the score functions to identify the native and near-native conformations among models with close-to-native conformation (most models deviate less than 6Å from the native X-ray structure), while *fisa_casp3 *and *lmds *sets are used to detect a small set of close to native conformations among many non-native conformers (most models deviate more than 5 Å from the native X-ray structure). We also checked that none of the sequences selected in these decoys were used on the construction of the statistical potentials.

### Scoring Functions

Several scoring functions (all of them based on statistical potentials) have been compared with ZE_min _and . The main difference between them lays on the definition of the reference state and in the composite of several scoring terms accounting for residue pair interactions and surface interactions.

*Prosa2003 *is a classical knowledge-based pair potential scoring function[66]. We have used *Prosa2003 *with default parameters. This implies the use of distance- and surface-dependent statistical potentials for C*β *atoms (C*α *for Gly) to calculate two different scores: a distance- dependent pair score and an accessible surface score. Both scores are combined into a score that has been used to test each model. The reference state is calculated with the total of observed pairs of residues.

*GA*_*341 *_is an optimized discriminator function[45] evolved by a genetic algorithm from a nonlinear combination of three model features and it includes a Zscore for the combined (distance and accessibility) residue-level statistical potential (obtained with the mean and standard deviation of the statistical potential score of 200 random sequences with the same amino acid residue-type composition and structure as the model).

Distance-scaled, Finite Ideal-gas REference (*DFIRE*) state is a scoring function[43] used to construct a residue specific all-atom potential of mean force from a database of protein structures with resolution less than 2 Å and less than 30% similarity between them. In this function, the equations from liquid-state statistical mechanics are modified for finite systems, like proteins, assuming that the expected number of contacts would not increase with r^2 ^but r^*α*^, where *α *is a tunable parameter optimized on the set of non-homologous proteins. The *DFIRE *program was used with default parameters (*α *= 1.57) to calculate the score for each model in the test set.

Similarly to *DFIRE*, another scoring function is defined as the Discrete Optimized Protein Energy (*DOPE*) approach[32]. This is a distance-dependant statistical potential based on an improved reference state that corresponds to non-interacting atoms in a homogeneous sphere that has to account for the finite size and spherical shape of proteins. A sample of many native structures of varying size is used to avoid the dependence of the scores between residues on the size of the protein.

### Statistical Analyses

We analyzed the use of scoring functions to predict the correct fold. On the one hand we used the scores to rank the conformations for each particular target within four decoy sets. This allowed us to test the ability on finding the right conformation within a set of putative models (i.e. the model with the first rank did coincide with the native structure of the target). On the other hand, thresholds were used to define positive/negative predictions: protein models with scores smaller than the threshold were predicted as positives and the remaining models were negatives. On the set of positives and negatives we defined the *true predictions *depending on the RMSD with respect to the native structure[64,65]. Among positives, true predictions (TP) were defined as those with RMSD smaller than 3Å with respect to the native structure and false predictions (FP) otherwise. Among negatives the inverted criterion was used, being false negatives (FN) those with RMSD smaller than 3Å and true negatives (TN) otherwise. Sensitivity or coverage was defined as the ratio of TP versus the total of true models (TP+FN). Specificity was defined as the ratio of TN/(TN+FP) and positive predictive value (PPV) as the ratio of TP/(TP+FP). Sensitivity, specificity and PPV were calculated for the 300 models of each target protein in MOULDER database.

First, the average and standard error of sensitivity, specificity and PPV calculated with the predictions of each 20 targets of MOULDER (i.e.  with x equal to sensitivity, specificity or PPV) were plotted versus the thresholds applied on the scores of several scoring methods. Second, all models from the 20 targets were used to calculate sensitivity, specificity and PPV versus these thresholds. While the first set of plots showed the ability of the score to detect the best conformation(s) (i.e. near-native conformations) among a pull of models generated with the same sequence, the second set of plots showed the ability to detect native and near-native folds among a pull of conformations with independence of its sequence. The threshold where sensitivity coincides with positive predictive value in the second set of plots is considered to be the best offset between coverage and PPV for each scoring method. These thresholds are used to calculate the distribution of RMSD, TP, FP, TN and FN for each scoring method in the set of MOULDER decoys. Finally, we plotted ROC curves of sensitivity/specificity and sensitivity/PPV calculated on MOULDER and *4state_reduced *decoy sets, because for *fisa_casp3 *and *lmds *sets the number of near-native conformations is small.

### Local conformation assessment

 and ZE_min _scores were used to check the local conformation. First, each residue was substituted by the remaining 19 possibilities (assuming that there are only 20 possible types of amino-acids) and the Zscores ( and ZE_min_) were recalculated. This produced 20 Zscores (one for the original amino-acid of the protein-sequence and 19 mutations for each position in the sequence) for  and ZE_min_. They were normalized with the 20 Zscores and they were transformed into single scores per residue-position named scanning-Zscores Sc and ScZE_min_, respectively. The normalization is obtained with the formulae: ScZE = (ZE-*μ*)/*σ*; where ZE is the corresponding Zscore with the original sequence ( and ZE_*min*_); *μ *is the average of the scores with the 19 substitutions plus the original sequence and *σ *the standard deviation. Second, a Zscore was calculated for each residue-position "*i*" by summing only the terms of equation 5 in which residue "*i*" participates (set Γ_*i *_in equation 5) and normalizing it into a Zscore with the energy terms of 1000 randomly shuffled sequences (see above). We obtained two Zscores for each residue-position from this second method (using C*β*-C*β *or *min *force-fields) that were named residue-position Zscores  and Z_A_E_min_, respectively.

## Authors' contributions

PA and BO conceived this work. PA provided the data, BO developed the software and both authors analyzed the results and wrote the manuscript. We also wish to thank the advise of our reviewers. All authors read and approved the final manuscript.

## Supplementary Material

Additional file 1 **Supplemental of Theory**. Derivation of equations and files used to train and test the statistical potentials. Click here for file

Additional file 2**Supplemental table S2: Differences of AUC and p-value of significance for scoring functions applied on MOULDER decoy sets**. Results obtained with the program StAR to assess the statistical significance of the observed difference between the scoring functions , ZE_min_, *DOPE, DFIRE, GA*_*341 *_and *Prosa2003 *when used as binary classifiers of the set of decoys of MOULDER. The upper right triangular part of the matrix shows the difference of the area under the curve of the ROC curves of true positive rate versus false positive rate. The lower left triangular part of the matrix shows the significant p-values of each pairwise comparison of classifiers (we assume that p-values smaller than 0.01 imply that the differences are significant). P-values higher than 0.01 are shown in red, and p-values between 0.01 and 0.001 in blue.Click here for file

Additional file 3**Supplemental table S3: Differences of AUC and p-value of significance for scoring functions applied on *4state_reduced *decoy sets**. Results obtained with the program StAR to assess the statistical significance of the observed difference between the scoring functions , ZE_min_, *DOPE, DFIRE, GA*_*341 *_and *Prosa2003 *when used as binary classifiers of the set of decoys of *4state_reduced*. The upper right triangular part of the matrix shows the difference of the area under the curve of the ROC curves of true positive rate versus false positive rate. The lower left triangular part of the matrix shows the significant p-values of each pairwise comparison of classifiers (p-values smaller than 0.001 imply that the differences are significant). P-values higher than 0.01 are shown in red, and p-values between 0.01 and 0.001 in blue.Click here for file

Additional file 4 **Supplemental table S1.** Averages and deviations of the *φ *parameters obtained with the C*β*-C*β *and *min *potentials. Results obtained for each subset on the 5-fold test are indicated in columns 1-fold, 2-fold, 3-fold, 4-fold and 5-fold. *Parameter optimization*: A total of 209 *φ *parameters are obtained for environment pairs expressed as a triad of polar character, secondary structure and exposure degree with *min *and C*β *potentials. Using a 5-fold procedure we obtain the average and standard deviation for each of them. About 15% of the parameters show less than 50% deviation, while around 50% show deviations larger than 100%. The largest percentages of deviation for C*β *potentials are obtained for [n-H-E:n-H-E] and [n-H-E:p-H-E], with more than 1000% deviation with respect to the average, while the largest deviation with the *min *potentials are for [p-C-E:p-E-B], [n-C-E:n-E-B] and [n-H-E:n-E-B], also with more than 1000% deviation. Among the most stable parameters, the minimum average values of C*β *potential and *min *potential are for [p-E-E:p-E-E] (-210 ± 66 kJ) and [n-E-E:n-E-E] (-210 ± 74 kJ), respectively. These large deviations imply that these parameters cannot be significant on the prediction of correct folds. This is in agreement with equation 2 (main text), where the term ^*ZE*^CMP was neglected (see text). Besides, the values cannot be used to further biological explanations, as they dramatically depend on the size and variability of data. Click here for file
