# Supplementary material for: Splitting statistical potentials into meaningful scoring functions: Testing the prediction of near-native structures from decoy conformations
Source: BMC Struct Biol. 2009 Nov 16;9:71. doi: 10.1186/1472-6807-9-71 (PMC2783033; doi:10.1186/1472-6807-9-71)
Supplement: Additional file 1 — Supplemental of Theory. Derivation of equations and files used to train and test the statistical potentials. [file 1472-6807-9-71-S1.DOC]

**Supplemental of Theory: Derivation of equations.**

The potential of mean force, formulae S1, is usually used to score the interaction between two residues. This score is obtained with the probability of finding a pair of residues (A_k_ and A_j_, with k and j between 1 and 20) at a distance “d” (i.e. P(A_k_,A_j_/d)), their individual probabilities on the database (ie. P(A_k_) and P(A_j_)) and the probability of any residue at this distance (i.e. P(d)) weighted by a specific reference state function (weight_ref_), where k_B_ is the Boltzmann constant and T the standard temperature (usually 300K).

(S1)

The *weight*_ref_ function can be defined as any function as long as it is positive, continuous and never close to 0, which allows the freedom for different definitions given by many authors.

Let be the sequence of a protein defined as the string: R_1_R_2_R_3_….R_N_

The score for a residue in position “i” (R_i_) is the total amount of its interactions with the rest of residues:

where Γ_i_ is the set of position-indexes of residues interacting with R_i_. The score for the fold is the sum of scores per amino-acid along the sequence.

We have derived a new scoring function that takes into account the conditions in which a residue is located in the structure. To define the conditions of a residue we used the polar character (n and p for non-polar and polar), secondary structure (H, E and C for helix, strand or coil) and degree of exposure (B and E for buried and exposed). These conditions were defined as triads of “hydrophobicity”-“secondary structure”-“exposure” (e.g. n-H-B identifies a residue non-polar, α-helix and buried). Equation S2 defines this potential with the conditional probabilities of residues A_k_ and A_j_ at distance d, being θ_n_ and θ_m_ their respective conditions. Indexes k and j are integers between 1 and 20, and indexes n and m are integers between 1 and 12.

(S2)

From equation S2 we can express the conditional probability of residues A_k_ and A_j_ being at distance *d* under conditions θ_n_ and θ_m_

(S3)

But we can also calculate this probability using the definition of conditional probability:

Hence, we can express P(A_k_,A_j_/d) as function of P(A_k_,A_j_/d ,θ_n_ , θ_m_):

(S4)

where also P(Ai,Aj/d) can be extracted from equation S1:

(S5)

By substituting equations S3 and S5 in S4 we obtain:

(S6)

Equation 6 can be reordered and simplified by using the following definitions:

(S7)

We wish to notice the difference between “PMF*_std_* (d)” in equations S1 and S2 and the new definitions of “PMF*std_NR*(d)” in S7, because the reference state function have been included in *w*_mn_. Equation S6 is rewritten as:

We try to solve the relationship between the original definition of knowledge-based potentials, PMF(A_k_,A_j_,d) and the sum of the new potential terms by adopting the hypothesis that there are two functions, ψ and ϕ, where: i) ψ is a function of the whole conformational space of protein-structures (PDB) with values 1 or 0, such that it is null unless the environments of the correct fold are given to a pair of interacting residues (and 1 otherwise); and ii) ϕ is a set of parameters dependent on the environments of each pair of interacting residues that includes the weights of the reference state, . This is:

When scoring a particular interaction at distance d between two residues, R_i_ and R_h_, under respective conditions θ_i_ and θ_h_ in a protein sequence, we obtain PMF(R_i_,R_h_,d). This could be rewritten under the previous hypothesis using equation S7 as:

(S8)

Finally the score for the fold of a protein is calculated by summing all residue-pair interactions. Therefore,

(S9)

And we define the new set of knowledge-based potentials in equation 1, to score the fold of a protein by the main terms appearing on equation S9:

(1)

Interestingly, we have transformed the problem of calculating the standard background energy (also named reference state) in two energetic terms in equation “1”: one is a fold dependent energy (E_3D_) and another has tunable parameters (E_REF_). These parameters can be calculated by optimizing the mean square error with respect to the original score calculated with the potential of mean force (i.e. using *PMF(R_i_,R_j_, d_ij_)* and 300K for the standard temperature T)

**List of SCOP families used on the training set (1764 domains)**

a.100.1

a.101.1

a.10.1

a.102.1

a.102.2

a.102.3

a.102.4

a.102.5

a.103.1

a.104.1

a.108.1

a.109.1

a.110.1

a.111.1

a.11.1

a.11.2

a.113.1

a.114.1

a.116.1

a.117.1

a.118.11

a.118.12

a.118.13

a.118.14

a.118.15

a.118.16

a.118.17

a.118.18

a.118.19

a.118.1

a.118.20

a.118.21

a.118.22

a.118.3

a.118.4

a.118.5

a.118.6

a.118.7

a.118.8

a.118.9

a.119.1

a.1.1

a.120.1

a.121.1

a.12.1

a.123.1

a.124.1

a.126.1

a.127.1

a.128.1

a.1.2

a.130.1

a.131.1

a.13.1

a.132.1

a.133.1

a.134.1

a.135.1

a.136.1

a.137.10

a.137.11

a.137.1

a.137.2

a.137.3

a.137.4

a.137.5

a.137.7

a.137.8

a.137.9

a.138.1

a.139.1

a.140.1

a.140.2

a.140.3

a.140.4

a.141.1

a.14.1

a.142.1

a.143.1

a.144.1

a.144.2

a.145.1

a.146.1

a.147.1

a.148.1

a.149.1

a.150.1

a.151.1

a.15.1

a.152.1

a.153.1

a.154.1

a.155.1

a.156.1

a.157.1

a.158.1

a.159.1

a.159.2

a.159.3

a.159.4

a.160.1

a.161.1

a.16.1

a.162.1

a.163.1

a.164.1

a.165.1

a.166.1

a.168.1

a.169.1

a.170.1

a.171.1

a.17.1

a.172.1

a.173.1

a.174.1

a.175.1

a.176.1

a.177.1

a.178.1

a.179.1

a.180.1

a.181.1

a.18.1

a.182.1

a.183.1

a.184.1

a.185.1

a.186.1

a.187.1

a.188.1

a.189.1

a.190.1

a.191.1

a.19.1

a.192.1

a.193.1

a.194.1

a.195.1

a.196.1

a.197.1

a.198.1

a.199.1

a.200.1

a.20.1

a.202.1

a.203.1

a.204.1

a.205.1

a.206.1

a.207.1

a.208.1

a.209.1

a.210.1

a.2.10

a.211.1

a.2.11

a.21.1

a.212.1

a.2.12

a.213.1

a.2.13

a.214.1

a.2.14

a.215.1

a.216.1

a.217.1

a.218.1

a.219.1

a.2.1

a.220.1

a.221.1

a.22.1

a.222.1

a.223.1

a.224.1

a.225.1

a.226.1

a.227.1

a.228.1

a.2.2

a.23.1

a.23.2

a.23.3

a.23.4

a.23.5

a.2.3

a.24.10

a.24.11

a.24.12

a.24.13

a.24.14

a.24.15

a.24.16

a.24.17

a.24.18

a.24.19

a.24.1

a.24.20

a.24.21

a.24.22

a.24.23

a.24.24

a.24.2

a.24.3

a.24.4

a.24.5

a.24.7

a.24.9

a.2.4

a.25.1

a.25.2

a.2.5

a.26.1

a.2.6

a.27.1

a.2.7

a.28.1

a.28.2

a.28.3

a.2.8

a.29.2

a.29.3

a.29.5

a.29.6

a.29.7

a.29.8

a.2.9

a.30.1

a.30.2

a.30.3

a.30.4

a.30.5

a.31.1

a.3.1

a.32.1

a.33.1

a.34.1

a.34.2

a.34.3

a.34.4

a.35.1

a.36.1

a.37.1

a.38.1

a.38.2

a.39.1

a.39.2

a.39.3

a.39.4

a.40.1

a.40.2

a.4.10

a.4.11

a.41.1

a.4.12

a.4.13

a.4.14

a.4.1

a.42.1

a.4.2

a.43.1

a.4.3

a.45.1

a.4.5

a.46.1

a.46.2

a.4.6

a.47.1

a.47.2

a.47.3

a.47.4

a.4.7

a.48.1

a.48.2

a.48.3

a.4.8

a.49.1

a.4.9

a.50.1

a.5.10

a.51.1

a.5.1

a.52.1

a.5.2

a.53.1

a.5.3

a.54.1

a.5.4

a.55.1

a.56.1

a.5.6

a.57.1

a.5.7

a.58.1

a.5.8

a.59.1

a.5.9

a.60.10

a.60.11

a.60.12

a.60.13

a.60.1

a.60.2

a.60.3

a.60.4

a.60.5

a.60.6

a.60.7

a.60.8

a.60.9

a.61.1

a.6.1

a.62.1

a.63.1

a.64.1

a.64.2

a.65.1

a.66.1

a.68.1

a.69.1

a.69.2

a.69.3

a.70.1

a.7.10

a.7.11

a.71.1

a.7.12

a.71.2

a.7.1

a.72.1

a.7.2

a.73.1

a.7.3

a.74.1

a.7.4

a.75.1

a.7.5

a.76.1

a.7.6

a.77.1

a.7.7

a.78.1

a.7.8

a.79.1

a.80.1

a.81.1

a.8.1

a.8.2

a.83.1

a.8.3

a.84.1

a.8.4

a.85.1

a.8.5

a.86.1

a.8.6

a.87.1

a.8.7

a.88.1

a.89.1

a.90.1

a.91.1

a.9.1

a.92.1

a.93.1

a.94.1

a.95.1

a.96.1

a.97.1

a.98.1

a.99.1

b.100.1

b.101.1

b.102.1

b.103.1

b.104.1

b.105.1

b.106.1

b.107.1

b.108.1

b.109.1

b.110.1

b.1.10

b.111.1

b.1.11

b.11.1

b.112.1

b.1.12

b.113.1

b.1.13

b.114.1

b.1.14

b.115.1

b.1.15

b.116.1

b.1.16

b.117.1

b.1.17

b.118.1

b.1.18

b.1.19

b.1.1

b.120.1

b.1.20

b.121.1

b.121.2

b.121.3

b.121.4

b.121.5

b.121.6

b.121.7

b.1.21

b.12.1

b.122.1

b.1.22

b.123.1

b.1.23

b.124.1

b.125.1

b.126.1

b.127.1

b.128.1

b.129.1

b.1.2

b.130.1

b.131.1

b.132.1

b.133.1

b.134.1

b.135.1

b.136.1

b.137.1

b.138.1

b.139.1

b.1.3

b.140.1

b.141.1

b.14.1

b.142.1

b.143.1

b.144.1

b.145.1

b.146.1

b.147.1

b.148.1

b.1.4

b.15.1

b.1.5

b.16.1

b.1.6

b.17.1

b.1.7

b.18.1

b.1.8

b.19.1

b.1.9

b.20.1

b.2.10

b.21.1

b.2.1

b.22.1

b.2.2

b.23.1

b.23.2

b.2.3

b.24.1

b.2.4

b.25.1

b.2.5

b.26.1

b.27.1

b.2.7

b.28.1

b.29.1

b.2.9

b.30.2

b.30.5

b.30.6

b.31.1

b.3.1

b.32.1

b.3.2

b.33.1

b.3.3

b.34.10

b.34.11

b.34.12

b.34.13

b.34.14

b.34.15

b.34.1

b.34.2

b.34.3

b.34.4

b.34.5

b.34.6

b.34.7

b.34.8

b.34.9

b.3.4

b.35.1

b.35.2

b.3.5

b.36.1

b.3.6

b.37.1

b.38.1

b.38.2

b.39.1

b.40.10

b.40.1

b.40.2

b.40.3

b.40.4

b.40.5

b.40.6

b.40.7

b.40.8

b.40.9

b.41.1

b.4.1

b.42.1

b.42.2

b.42.3

b.42.4

b.42.5

b.42.6

b.42.7

b.42.8

b.43.2

b.43.3

b.43.4

b.43.5

b.44.1

b.44.2

b.45.1

b.46.1

b.47.1

b.48.1

b.49.1

b.49.3

b.50.1

b.51.1

b.5.1

b.52.1

b.52.2

b.53.1

b.54.1

b.55.1

b.56.1

b.57.1

b.58.1

b.59.1

b.60.1

b.61.1

b.61.2

b.61.3

b.61.4

b.61.5

b.61.6

b.6.1

b.62.1

b.6.2

b.63.1

b.64.1

b.65.1

b.66.1

b.67.1

b.67.2

b.67.3

b.68.10

b.68.1

b.68.2

b.68.3

b.68.4

b.68.5

b.68.6

b.68.7

b.68.8

b.68.9

b.69.10

b.69.11

b.69.12

b.69.13

b.69.1

b.69.2

b.69.3

b.69.4

b.69.5

b.69.6

b.69.7

b.69.8

b.69.9

b.70.1

b.70.2

b.70.3

b.71.1

b.7.1

b.72.1

b.72.2

b.72.3

b.7.2

b.73.1

b.7.3

b.74.1

b.7.4

b.75.1

b.76.1

b.76.2

b.77.1

b.77.2

b.77.3

b.78.1

b.80.1

b.80.2

b.80.3

b.80.4

b.80.5

b.80.6

b.80.7

b.81.1

b.81.2

b.81.3

b.8.1

b.82.1

b.82.2

b.82.3

b.82.4

b.82.5

b.82.6

b.82.7

b.83.1

b.84.1

b.84.2

b.84.3

b.84.4

b.85.1

b.85.2

b.85.3

b.85.4

b.85.5

b.85.6

b.85.7

b.86.1

b.87.1

b.88.1

b.89.1

b.90.1

b.91.1

b.9.1

b.92.1

b.93.1

b.94.1

b.95.1

b.96.1

b.97.1

b.98.1

c.100.1

c.101.1

c.10.1

c.102.1

c.10.2

c.103.1

c.10.3

c.104.1

c.105.1

c.106.1

c.107.1

c.108.1

c.109.1

c.110.1

c.1.10

c.111.1

c.1.11

c.112.1

c.1.12

c.113.1

c.1.13

c.114.1

c.1.14

c.115.1

c.1.15

c.116.1

c.1.16

c.117.1

c.1.17

c.118.1

c.1.18

c.119.1

c.1.19

c.1.1

c.120.1

c.1.20

c.121.1

c.1.21

c.12.1

c.122.1

c.1.22

c.123.1

c.1.23

c.124.1

c.1.24

c.125.1

c.1.25

c.126.1

c.1.26

c.127.1

c.1.27

c.128.1

c.1.28

c.129.1

c.1.29

c.1.2

c.130.1

c.1.30

c.131.1

c.1.31

c.13.1

c.132.1

c.13.2

c.133.1

c.134.1

c.135.1

c.136.1

c.137.1

c.138.1

c.139.1

c.1.3

c.140.1

c.141.1

c.14.1

c.1.4

c.15.1

c.1.5

c.16.1

c.1.6

c.17.1

c.1.7

c.18.1

c.1.8

c.19.1

c.1.9

c.20.1

c.21.1

c.2.1

c.22.1

c.23.10

c.23.11

c.23.13

c.23.14

c.23.15

c.23.16

c.23.17

c.23.1

c.23.2

c.23.3

c.23.4

c.23.5

c.23.6

c.23.8

c.24.1

c.25.1

c.26.1

c.26.2

c.26.3

c.27.1

c.28.1

c.30.1

c.31.1

c.3.1

c.32.1

c.33.1

c.34.1

c.36.1

c.37.1

c.38.1

c.39.1

c.40.1

c.41.1

c.42.1

c.43.1

c.44.1

c.44.2

c.45.1

c.46.1

c.47.1

c.47.2

c.48.1

c.49.1

c.49.2

c.50.1

c.51.1

c.51.2

c.51.3

c.51.4

c.5.1

c.52.1

c.52.2

c.52.3

c.53.1

c.53.2

c.54.1

c.55.1

c.55.2

c.55.3

c.55.4

c.55.5

c.55.6

c.55.7

c.56.1

c.56.2

c.56.3

c.56.4

c.56.5

c.56.6

c.57.1

c.58.1

c.59.1

c.60.1

c.61.1

c.6.1

c.62.1

c.6.2

c.6.3

c.64.1

c.65.1

c.66.1

c.67.1

c.68.1

c.69.1

c.70.1

c.71.1

c.7.1

c.72.1

c.72.2

c.72.3

c.73.1

c.74.1

c.76.1

c.77.1

c.78.1

c.78.2

c.79.1

c.80.1

c.81.1

c.8.1

c.82.1

c.8.2

c.83.1

c.8.3

c.84.1

c.8.4

c.85.1

c.8.5

c.86.1

c.8.6

c.87.1

c.8.7

c.88.1

c.8.8

c.89.1

c.90.1

c.91.1

c.9.1

c.92.1

c.92.2

c.9.2

c.93.1

c.94.1

c.95.1

c.96.1

c.97.1

c.97.2

c.97.3

c.98.1

c.98.2

c.99.1

d.100.1

d.101.1

d.10.1

d.102.1

d.103.1

d.104.1

d.105.1

d.106.1

d.107.1

d.108.1

d.109.1

d.109.2

d.110.1

d.110.2

d.110.3

d.110.4

d.110.5

d.110.6

d.110.7

d.111.1

d.11.1

d.112.1

d.113.1

d.114.1

d.115.1

d.116.1

d.117.1

d.118.1

d.1.1

d.120.1

d.121.1

d.12.1

d.122.1

d.123.1

d.124.1

d.125.1

d.126.1

d.127.1

d.128.1

d.129.1

d.129.2

d.129.3

d.129.4

d.129.5

d.129.6

d.129.7

d.129.8

d.130.1

d.131.1

d.13.1

d.13.2

d.133.1

d.134.1

d.135.1

d.136.1

d.137.1

d.138.1

d.139.1

d.140.1

d.141.1

d.14.1

d.142.1

d.142.2

d.143.1

d.144.1

d.145.1

d.146.1

d.147.1

d.148.1

d.149.1

d.150.1

d.15.10

d.15.11

d.151.1

d.15.12

d.15.1

d.152.1

d.15.2

d.153.1

d.153.2

d.15.3

d.154.1

d.15.4

d.155.1

d.15.5

d.156.1

d.15.6

d.157.1

d.15.7

d.15.8

d.159.1

d.15.9

d.160.1

d.161.1

d.16.1

d.162.1

d.163.1

d.164.1

d.165.1

d.166.1

d.167.1

d.168.1

d.169.1

d.170.1

d.170.2

d.171.1

d.17.1

d.172.1

d.17.2

d.173.1

d.17.3

d.174.1

d.17.4

d.175.1

d.17.5

d.176.1

d.17.6

d.177.1

d.17.7

d.178.1

d.180.1

d.181.1

d.18.1

d.182.1

d.183.1

d.184.1

d.185.1

d.186.1

d.187.1

d.188.1

d.189.1

d.190.1

d.19.1

d.192.1

d.193.1

d.194.1

d.195.1

d.196.1

d.197.1

d.198.1

d.198.2

d.199.1

d.200.1

d.201.1

d.20.1

d.202.1

d.203.1

d.204.1

d.205.1

d.206.1

d.207.1

d.208.1

d.209.1

d.210.1

d.211.1

d.211.2

d.21.1

d.212.1

d.213.1

d.214.1

d.215.1

d.216.1

d.217.1

d.218.1

d.219.1

d.2.1

d.220.1

d.221.1

d.22.1

d.222.1

d.223.1

d.224.1

d.225.1

d.226.1

d.227.1

d.228.1

d.229.1

d.230.1

d.230.2

d.230.3

d.231.1

d.23.1

d.232.1

d.233.1

d.234.1

d.235.1

d.236.1

d.237.1

d.238.1

d.239.1

d.240.1

d.241.1

d.241.2

d.24.1

d.242.1

d.243.1

d.244.1

d.245.1

d.246.1

d.247.1

d.248.1

d.249.1

d.250.1

d.251.1

d.25.1

d.252.1

d.253.1

d.254.1

d.255.1

d.256.1

d.257.1

d.258.1

d.259.1

d.260.1

d.261.1

d.26.1

d.262.1

d.26.2

d.263.1

d.26.3

d.264.1

d.265.1

d.266.1

d.267.1

d.268.1

d.269.1

d.270.1

d.271.1

d.27.1

d.272.1

d.273.1

d.274.1

d.275.1

d.276.1

d.277.1

d.278.1

d.279.1

d.280.1

d.281.1

d.28.1

d.282.1

d.283.1

d.284.1

d.285.1

d.29.1

d.30.1

d.31.1

d.3.1

d.32.1

d.33.1

d.34.1

d.35.1

d.36.1

d.37.1

d.38.1

d.39.1

d.40.1

d.41.1

d.41.2

d.41.3

d.41.4

d.41.5

d.4.1

d.42.1

d.43.1

d.44.1

d.45.1

d.47.1

d.48.1

d.48.2

d.49.1

d.50.1

d.50.2

d.50.3

d.50.4

d.51.1

d.5.1

d.52.1

d.52.2

d.52.3

d.52.4

d.52.5

d.52.6

d.52.7

d.53.1

d.54.1

d.55.1

d.57.1

d.58.10

d.58.11

d.58.12

d.58.13

d.58.14

d.58.15

d.58.16

d.58.17

d.58.18

d.58.19

d.58.1

d.58.20

d.58.21

d.58.22

d.58.23

d.58.24

d.58.25

d.58.26

d.58.27

d.58.28

d.58.29

d.58.2

d.58.30

d.58.31

d.58.32

d.58.33

d.58.34

d.58.36

d.58.38

d.58.39

d.58.3

d.58.40

d.58.41

d.58.42

d.58.43

d.58.44

d.58.46

d.58.47

d.58.48

d.58.49

d.58.4

d.58.50

d.58.51

d.58.52

d.58.5

d.58.6

d.58.7

d.58.8

d.58.9

d.59.1

d.60.1

d.61.1

d.6.1

d.62.1

d.63.1

d.64.1

d.65.1

d.66.1

d.67.1

d.67.2

d.67.3

d.67.4

d.68.1

d.68.2

d.68.3

d.68.4

d.68.5

d.68.6

d.68.7

d.70.1

d.71.1

d.7.1

d.72.1

d.73.1

d.74.1

d.74.2

d.74.3

d.74.4

d.75.1

d.75.2

d.76.1

d.77.1

d.78.1

d.79.1

d.79.2

d.79.3

d.79.4

d.79.5

d.79.6

d.79.7

d.80.1

d.81.1

d.8.1

d.82.1

d.82.2

d.82.3

d.83.1

d.83.2

d.84.1

d.85.1

d.86.1

d.87.1

d.87.2

d.88.1

d.89.1

d.90.1

d.91.1

d.9.1

d.92.1

d.92.2

d.93.1

d.94.1

d.94.2

d.95.1

d.95.2

d.96.1

d.97.1

d.98.1

d.99.1

e.10.1

e.11.1

e.1.1

e.12.1

e.13.1

e.15.1

e.17.1

e.18.1

e.19.1

e.2.1

e.22.1

e.23.1

e.24.1

e.25.1

e.26.1

e.27.1

e.28.1

e.29.1

e.3.1

e.32.1

e.34.1

e.37.1

e.38.1

e.39.1

e.40.1

e.41.1

e.4.1

e.42.1

e.43.1

e.44.1

e.45.1

e.46.1

e.47.1

e.48.1

e.49.1

e.50.1

e.51.1

e.5.1

e.52.1

e.53.1

e.54.1

e.55.1

e.6.1

e.7.1

e.8.1

f.10.1

f.11.1

f.1.1

f.1.2

f.13.1

f.1.3

f.14.1

f.1.4

f.15.1

f.1.5

f.16.1

f.17.1

f.17.2

f.18.1

f.19.1

f.20.1

f.21.1

f.21.2

f.21.3

f.22.1

f.23.10

f.23.11

f.23.12

f.23.13

f.23.14

f.23.15

f.23.16

f.23.17

f.23.18

f.23.19

f.23.1

f.23.20

f.23.21

f.23.22

f.23.23

f.23.24

f.23.25

f.23.26

f.23.27

f.23.28

f.23.29

f.23.2

f.23.30

f.23.3

f.23.4

f.23.5

f.23.6

f.23.7

f.23.8

f.23.9

f.24.1

f.25.1

f.26.1

f.27.1

f.28.1

f.29.1

f.30.1

f.31.1

f.3.1

f.32.1

f.34.1

f.36.1

f.37.1

f.38.1

f.39.1

f.40.1

f.41.1

f.4.1

f.42.1

f.4.2

f.43.1

f.4.3

f.44.1

f.4.4

f.45.1

f.4.5

f.46.1

f.4.6

f.47.1

f.48.1

f.5.1

f.6.1

f.7.1

f.8.1

f.9.1

g.10.1

g.11.1

g.1.1

g.12.1

g.13.1

g.14.1

g.16.1

g.16.2

g.17.1

g.18.1

g.19.1

g.20.1

g.21.1

g.2.1

g.22.1

g.2.2

g.23.1

g.2.3

g.24.1

g.25.1

g.26.1

g.27.1

g.28.1

g.29.1

g.30.1

g.3.10

g.3.11

g.31.1

g.3.12

g.3.13

g.3.14

g.3.15

g.3.16

g.3.17

g.3.18

g.3.19

g.3.1

g.32.1

g.3.2

g.33.1

g.3.3

g.34.1

g.3.4

g.35.1

g.3.5

g.36.1

g.3.6

g.37.1

g.3.7

g.38.1

g.3.8

g.39.1

g.3.9

g.40.1

g.41.10

g.41.11

g.41.12

g.41.13

g.41.1

g.41.2

g.41.3

g.41.4

g.41.5

g.41.6

g.41.7

g.41.8

g.41.9

g.4.1

g.42.1

g.43.1

g.44.1

g.45.1

g.46.1

g.47.1

g.48.1

g.49.1

g.50.1

g.51.1

g.5.1

g.52.1

g.53.1

g.54.1

g.55.1

g.58.1

g.59.1

g.60.1

g.61.1

g.6.1

g.62.1

g.63.1

g.64.1

g.65.1

g.66.1

g.67.1

g.68.1

g.69.1

g.70.1

g.71.1

g.7.1

g.72.1

g.73.1

g.74.1

g.75.1

g.76.1

g.77.1

g.8.1

g.9.1

h.1.10

h.1.11

h.1.12

h.1.13

h.1.14

h.1.15

h.1.16

h.1.17

h.1.18

h.1.19

h.1.1

h.1.20

h.1.21

h.1.22

h.1.23

h.1.24

h.1.25

h.1.26

h.1.27

h.1.28

h.1.2

h.1.3

h.1.4

h.1.5

h.1.6

h.1.7

h.1.8

h.1.9

h.2.1

h.3.1

h.3.2

h.3.3

h.4.10

h.4.11

h.4.12

h.4.13

h.4.14

h.4.15

h.4.16

h.4.1

h.4.2

h.4.3

h.4.4

h.4.5

h.4.6

h.4.7

h.4.8

h.4.9

h.5.1

h.6.1

i.10.1

i.11.1

i.1.1

i.12.1

i.13.1

i.14.1

i.15.1

i.16.1

i.17.1

i.18.1

i.19.1

i.20.1

i.21.1

i.2.1

i.22.1

i.23.1

i.24.1

i.3.1

i.4.1

i.5.1

i.6.1

i.7.1

i.8.1

i.9.1

j.100.1

j.101.1

j.10.1

j.102.1

j.103.1

j.104.1

j.105.1

j.106.1

j.107.1

j.108.1

j.109.1

j.110.1

j.111.1

j.11.1

j.112.1

j.113.1

j.114.1

j.115.1

j.116.1

j.117.1

j.1.1

j.12.1

j.13.1

j.14.1

j.15.1

j.16.1

j.17.1

j.18.1

j.19.1

j.20.1

j.21.1

j.2.1

j.22.1

j.23.1

j.24.1

j.25.1

j.26.1

j.27.1

j.28.1

j.29.1

j.30.1

j.31.1

j.3.1

j.32.1

j.33.1

j.33.2

j.34.1

j.35.1

j.36.1

j.36.2

j.36.3

j.36.4

j.37.1

j.38.1

j.39.1

j.40.1

j.41.1

j.4.1

j.42.1

j.43.1

j.44.1

j.45.1

j.47.1

j.48.1

j.49.1

j.50.1

j.51.1

j.5.1

j.52.1

j.53.1

j.54.1

j.55.1

j.56.1

j.57.1

j.58.1

j.59.1

j.60.1

j.61.1

j.6.1

j.62.1

j.63.1

j.64.1

j.65.1

j.66.1

j.67.1

j.68.1

j.69.1

j.70.1

j.71.1

j.7.1

j.72.1

j.7.2

j.73.1

j.74.1

j.75.1

j.76.1

j.77.1

j.78.1

j.79.1

j.80.1

j.81.1

j.8.1

j.82.1

j.83.1

j.84.1

j.85.1

j.86.1

j.87.1

j.88.1

j.89.1

j.90.1

j.91.1

j.9.1

j.92.1

j.9.2

j.93.1

j.9.3

j.94.1

j.9.4

j.95.1

j.9.5

j.96.1

j.9.6

j.97.1

j.98.1

j.99.1

k.10.1

k.11.1

k.1.1

k.12.1

k.13.1

k.14.1

k.15.1

k.16.1

k.17.1

k.18.1

k.19.1

k.20.1

k.21.1

k.2.1

k.22.1

k.23.1

k.24.1

k.26.1

k.27.1

k.28.1

k.29.1

k.30.1

k.31.1

k.3.1

k.32.1

k.33.1

k.34.1

k.35.1

k.36.1

k.37.1

k.38.1

k.39.1

k.40.1

k.41.1

k.4.1

k.42.1

k.43.1

k.44.1

k.6.1

k.7.1

k.8.1

k.9.1
