# Supplementary material for: Splitting statistical potentials into meaningful scoring functions: Testing the prediction of near-native structures from decoy conformations
Source: BMC Struct Biol. 2009 Nov 16;9:71. doi: 10.1186/1472-6807-9-71 (PMC2783033; doi:10.1186/1472-6807-9-71)
Supplement: Additional file 2 — Supplemental table S2: Differences of AUC and p-value of significance for scoring functions applied on MOULDER decoy sets. Results obtained with the program StAR to assess the statistical significance of the observed difference between the scoring functions , ZEmin, DOPE, DFIRE, GA341 and Prosa2003 when used as binary classifiers of the set of decoys of MOULDER. The upper right triangular part of the matrix shows the difference of the area under the curve of the ROC curves of true positive rate versus false positive rate. The lower left triangular part of the matrix shows the significant p-values of each pairwise comparison of classifiers (we assume that p-values smaller than 0.01 imply that the differences are significant). P-values higher than 0.01 are shown in red, and p-values between 0.01 and 0.001 in blue. [file 1472-6807-9-71-S2.DOC]

**Supplemental table S.2. Differences of AUC and p-value of significance for scoring functions applied on MOULDER decoy sets.** Results obtained with the program StAR to assess the statistical significance of the observed difference between the scoring functions ZE_Cβ_, ZE_min_, *DOPE, DFIRE, GA_341_* and *Prosa2003* when used as binary classifiers of the set of decoys of MOULDER. The upper right triangular part of the matrix shows the difference of the area under the curve of the ROC curves of true positive rate versus false positive rate. The lower left triangular part of the matrix shows the significant p-values of each pairwise comparison of classifiers (we assume that p-values smaller than 0.01 imply that the differences are significant). P-values higher than 0.01 are shown in red, and p-values between 0.01 and 0.001 in blue.

|  | *GA_341_* | *Prosa2003* | *DFIRE* | *DOPE* | ZE_Cβ_ | ZE_min_ |
| --- | --- | --- | --- | --- | --- | --- |
| *GA_341_* |  | 1,48E-01 | 3,04E-02 | 1,45E-02 | 1,18E-02 | 5,33E-03 |
| *Prosa2003* | 1,60E-33 |  | 1,78E-01 | 1,62E-01 | 1,59E-01 | 1,53E-01 |
| *DFIRE* | 7,86E-08 | 1,93E-43 |  | 1,58E-02 | 1,86E-02 | 2,51E-02 |
| *DOPE* | 5,07E-02 | 1,29E-33 | 3,94E-05 |  | 2,79E-03 | 9,21E-03 |
| ZE_Cβ_ | 3,64E-02 | 2,98E-36 | 1,19E-03 | 7,03E-01 |  | 6,43E-03 |
| ZE_min_ | 4,36E-01 | 1,97E-29 | 6,75E-04 | 2,22E-01 | 3,45E-01 |  |
