# Supplementary material for: Splitting statistical potentials into meaningful scoring functions: Testing the prediction of near-native structures from decoy conformations
Source: BMC Struct Biol. 2009 Nov 16;9:71. doi: 10.1186/1472-6807-9-71 (PMC2783033; doi:10.1186/1472-6807-9-71)
Supplement: Additional file 4 — Supplemental table S1. Averages and deviations of the φ parameters obtained with the Cβ-Cβ and min potentials. Results obtained for each subset on the 5-fold test are indicated in columns 1-fold, 2-fold, 3-fold, 4-fold and 5-fold. Parameter optimization: A total of 209 φ parameters are obtained for environment pairs expressed as a triad of polar character, secondary structure and exposure degree with min and Cβ potentials. Using a 5-fold procedure we obtain the average and standard deviation for each of them. About 15% of the parameters show less than 50% deviation, while around 50% show deviations larger than 100%. The largest percentages of deviation for Cβ potentials are obtained for [n-H-E:n-H-E] and [n-H-E:p-H-E], with more than 1000% deviation with respect to the average, while the largest deviation with the min potentials are for [p-C-E:p-E-B], [n-C-E:n-E-B] and [n-H-E:n-E-B], also with more than 1000% deviation. Among the most stable parameters, the minimum average values of Cβ potential and min potential are for [p-E-E:p-E-E] (-210 ± 66 kJ) and [n-E-E:n-E-E] (-210 ± 74 kJ), respectively. These large deviations imply that these parameters cannot be significant on the prediction of correct folds. This is in agreement with equation 2 (main text), where the term ZECMP was neglected (see text). Besides, the values cannot be used to further biological explanations, as they dramatically depend on the size and variability of data. [file 1472-6807-9-71-S4.DOC]

**Supplemental table S.1. Averages and deviations of the ϕ parameters obtained with the Cβ−Cβ and *min* potentials.**  Results obtained for each subset on the 5-fold test are indicated in columns 1-fold, 2-fold, 3-fold, 4-fold and 5-fold.

*Parameter optimization*

A total of 209 *ϕ *parameters are obtained for environment pairs expressed as a triad of polar character, secondary structure and exposure degree with *min* and Cβ potentials. Using a 5-fold procedure we obtain the average and standard deviation for each of them. About 15 % of the parameters show less than 50% deviation, while around 50% show deviations larger than 100% (table S.1). The largest percentages of deviation for Cβ potentials are obtained for [n-H-E:n-H-E] and [n-H-E:p-H-E], with more than 1000% deviation with respect to the average, while the largest deviation with the *min* potentials are for [p-C-E:p-E-B], [n-C-E:n-E-B] and [n-H-E:n-E-B], also with more than 1000% deviation. Among the most stable parameters, the minimum average values of Cβ potential and *min* potential are for [p-E-E:p-E-E] (-210±66kJ) and [n-E-E:n-E-E] (-210±74kJ), respectively. These large deviations imply that these parameters cannot be significant on the prediction of correct folds. This is in agreement with equation 2 (main text), where the term ZE_CMP_ was neglected (see text). Besides, the values cannot be used to further biological explanations, as they dramatically depend on the size and variability of data.

| ϕ parameter Cβ−Cβ | 1-fold | 2-fold | 3-fold | 4-fold | 5-fold | Average | RMSD | % RMSD |
| --- | --- | --- | --- | --- | --- | --- | --- | --- |
| p-E-E:p-E-E | -1.7e+02 | -3.1e+02 | -2.7e+02 | -1.9e+02 | -1.3e+02 | -2.1e+02 | 6.6e+01 | 3.1e+01 |
| n-E-E:n-E-E | -1.7e+02 | 1.7e+01 | -1.9e+02 | -1.7e+02 | -2.1e+02 | -1.5e+02 | 8.3e+01 | 5.7e+01 |
| p-E-E:n-H-E | -1.0e+02 | -1.2e+02 | -2.3e+02 | -1.5e+02 | 0.0e+00 | -1.2e+02 | 7.3e+01 | 6.1e+01 |
| n-E-E:p-H-E | -5.2e+02 | 0.0e+00 | 0.0e+00 | 0.0e+00 | 0.0e+00 | -1.0e+02 | 2.1e+02 | 2.0e+02 |
| p-H-E:n-E-E | 0.0e+00 | -1.1e+02 | -3.0e+02 | -1.9e+02 | 1.7e+02 | -8.8e+01 | 1.6e+02 | 1.8e+02 |
| n-C-E:p-E-E | -7.3e+01 | -4.8e+01 | -6.1e+01 | -9.3e+00 | -6.1e+01 | -5.0e+01 | 2.2e+01 | 4.4e+01 |
| n-H-B:p-E-E | -5.7e+01 | -4.4e+01 | -2.0e+01 | -8.9e+01 | -3.3e+01 | -4.8e+01 | 2.4e+01 | 4.9e+01 |
| n-H-E:p-E-E | 0.0e+00 | 0.0e+00 | 0.0e+00 | 0.0e+00 | -2.3e+02 | -4.7e+01 | 9.4e+01 | 2.0e+02 |
| n-C-E:n-H-E | -3.5e+01 | -1.2e+01 | -1.3e+01 | -2.7e+01 | -5.2e+01 | -2.8e+01 | 1.5e+01 | 5.4e+01 |
| p-H-B:p-E-E | -9.7e-01 | 1.2e+01 | -6.9e+01 | -4.8e+01 | -2.3e+01 | -2.6e+01 | 3.0e+01 | 1.1e+02 |
| n-E-B:n-E-E | -3.3e+01 | -1.3e+01 | 0.0e+00 | -3.6e+01 | -2.9e+01 | -2.2e+01 | 1.4e+01 | 6.1e+01 |
| n-C-E:p-H-E | -2.9e+01 | -1.5e+01 | -8.9e+00 | -1.1e+01 | -2.8e+01 | -1.8e+01 | 8.4e+00 | 4.6e+01 |
| p-C-E:p-E-E | 2.6e+01 | -1.0e+01 | -4.0e+01 | -5.7e+01 | 0.0e+00 | -1.6e+01 | 2.9e+01 | 1.8e+02 |
| p-C-B:n-E-E | -4.4e+01 | 7.7e+00 | -2.9e+01 | -1.2e+01 | 8.1e+00 | -1.4e+01 | 2.1e+01 | 1.5e+02 |
| p-E-E:p-E-B | -1.7e+01 | -1.0e+01 | -1.3e+01 | -2.4e+01 | -7.7e+00 | -1.4e+01 | 5.6e+00 | 3.9e+01 |
| p-E-B:n-C-E | -1.4e+01 | -1.2e+01 | 0.0e+00 | -1.6e+01 | -1.6e+01 | -1.1e+01 | 5.9e+00 | 5.2e+01 |
| p-H-E:p-H-E | -6.5e+00 | -2.3e+01 | 2.7e+00 | -1.3e+01 | -8.9e+00 | -9.6e+00 | 8.3e+00 | 8.6e+01 |
| p-E-E:n-C-B | -9.7e+00 | -2.3e+01 | 0.0e+00 | -1.4e+01 | 7.3e+00 | -8.0e+00 | 1.1e+01 | 1.3e+02 |
| n-H-E:p-C-B | 3.5e+00 | -1.3e+01 | -1.3e+01 | -5.2e+00 | -1.0e+01 | -7.5e+00 | 6.2e+00 | 8.2e+01 |
| p-E-E:p-C-E | 0.0e+00 | 0.0e+00 | 0.0e+00 | 0.0e+00 | -3.4e+01 | -6.9e+00 | 1.4e+01 | 2.0e+02 |
| p-C-E:p-C-E | -1.6e+00 | 2.1e+00 | -7.7e+00 | -1.5e+01 | -6.9e+00 | -5.9e+00 | 5.9e+00 | 1.0e+02 |
| n-E-E:n-E-B | 0.0e+00 | 0.0e+00 | -2.9e+01 | 0.0e+00 | 0.0e+00 | -5.7e+00 | 1.1e+01 | 2.0e+02 |
| n-E-B:p-H-E | -1.1e+01 | 1.5e+01 | -2.3e+01 | 0.0e+00 | -8.1e+00 | -5.2e+00 | 1.3e+01 | 2.4e+02 |
| p-H-B:n-H-E | -6.1e+00 | 0.0e+00 | -8.1e+00 | -3.3e+00 | -5.2e+00 | -4.5e+00 | 2.7e+00 | 6.0e+01 |
| p-C-B:n-H-B | -5.7e+00 | 0.0e+00 | -5.2e+00 | -5.2e+00 | -3.0e+00 | -3.8e+00 | 2.1e+00 | 5.6e+01 |
| p-C-B:n-C-B | -4.0e+00 | 0.0e+00 | -3.6e+00 | -4.8e+00 | -4.8e+00 | -3.5e+00 | 1.8e+00 | 5.2e+01 |
| n-E-B:p-C-B | -1.5e+00 | -7.3e-01 | -4.4e+00 | -5.2e+00 | -4.4e+00 | -3.3e+00 | 1.8e+00 | 5.5e+01 |
| p-H-B:n-C-E | -1.7e-01 | 0.0e+00 | -4.0e+00 | -5.2e+00 | -6.5e+00 | -3.2e+00 | 2.6e+00 | 8.3e+01 |
| n-E-B:p-E-B | -2.9e+00 | -4.8e+00 | -1.4e+00 | -2.6e+00 | -3.7e+00 | -3.1e+00 | 1.2e+00 | 3.8e+01 |
| n-C-E:p-E-B | 0.0e+00 | 0.0e+00 | -1.5e+01 | 0.0e+00 | 0.0e+00 | -3.0e+00 | 6.0e+00 | 2.0e+02 |
| p-C-B:p-C-B | -1.5e+00 | -1.6e+00 | -4.4e+00 | -2.5e+00 | -4.0e+00 | -2.8e+00 | 1.2e+00 | 4.4e+01 |
| n-C-B:p-E-B | -2.7e-02 | -3.6e+00 | -1.9e+00 | -2.9e+00 | -4.8e+00 | -2.7e+00 | 1.6e+00 | 6.1e+01 |
| n-H-B:n-C-B | -4.4e+00 | -1.6e+00 | -2.6e+00 | -2.7e+00 | -2.0e+00 | -2.7e+00 | 9.8e-01 | 3.7e+01 |
| n-C-B:n-H-E | -1.3e+01 | 0.0e+00 | 0.0e+00 | 0.0e+00 | 0.0e+00 | -2.6e+00 | 5.2e+00 | 2.0e+02 |
| p-C-E:p-E-B | -5.2e+00 | -9.3e-01 | -1.3e+00 | 0.0e+00 | -4.8e+00 | -2.5e+00 | 2.2e+00 | 8.8e+01 |
| n-H-B:n-C-E | 3.3e-01 | 6.5e+00 | -3.3e+00 | -8.5e+00 | -6.9e+00 | -2.4e+00 | 5.4e+00 | 2.3e+02 |
| n-H-B:p-E-B | -6.1e-01 | -3.5e+00 | -3.7e+00 | -4.8e-01 | -3.5e+00 | -2.4e+00 | 1.5e+00 | 6.3e+01 |
| p-E-E:p-C-B | -8.9e+00 | -6.1e+00 | 0.0e+00 | 1.5e+01 | -1.2e+01 | -2.4e+00 | 9.3e+00 | 3.8e+02 |
| n-H-B:n-E-B | -1.2e+00 | -1.8e+00 | 0.0e+00 | -3.1e+00 | -5.2e+00 | -2.3e+00 | 1.8e+00 | 7.9e+01 |
| n-H-E:n-H-E | 1.9e+01 | -4.4e+01 | -2.7e+01 | 1.7e+01 | 2.4e+01 | -2.3e+00 | 2.8e+01 | 1.2e+03 |
| n-C-E:n-E-B | 0.0e+00 | 0.0e+00 | -1.1e+01 | 0.0e+00 | 0.0e+00 | -2.2e+00 | 4.4e+00 | 2.0e+02 |
| n-C-B:n-C-B | -2.4e+00 | -2.1e+00 | -8.9e-02 | -2.1e+00 | -3.4e+00 | -2.0e+00 | 1.1e+00 | 5.3e+01 |
| p-C-B:p-E-B | 7.3e-01 | -6.5e-01 | -2.1e+00 | -2.5e+00 | -3.3e+00 | -1.6e+00 | 1.4e+00 | 9.1e+01 |
| n-H-B:n-H-B | -1.3e+00 | -1.2e+00 | -2.0e+00 | -3.4e+00 | 2.7e-01 | -1.5e+00 | 1.2e+00 | 7.9e+01 |
| p-H-E:p-C-B | -6.1e-01 | 9.3e+00 | -6.5e+00 | -7.3e+00 | -2.4e+00 | -1.5e+00 | 5.9e+00 | 4.0e+02 |
| p-H-E:n-E-B | 0.0e+00 | 0.0e+00 | 0.0e+00 | -6.9e+00 | 0.0e+00 | -1.4e+00 | 2.7e+00 | 2.0e+02 |
| n-E-B:n-E-B | -1.0e+00 | -1.3e+00 | -1.3e+00 | -2.3e+00 | -6.5e-01 | -1.3e+00 | 5.4e-01 | 4.1e+01 |
| n-C-E:p-H-B | 0.0e+00 | -5.7e+00 | 0.0e+00 | 0.0e+00 | 0.0e+00 | -1.1e+00 | 2.3e+00 | 2.0e+02 |
| n-H-B:p-C-B | 0.0e+00 | -4.8e+00 | 0.0e+00 | 0.0e+00 | 0.0e+00 | -9.7e-01 | 1.9e+00 | 2.0e+02 |
| p-C-B:n-C-E | 0.0e+00 | -4.8e+00 | 0.0e+00 | 0.0e+00 | 0.0e+00 | -9.7e-01 | 1.9e+00 | 2.0e+02 |
| p-H-B:n-H-B | -1.0e+00 | 0.0e+00 | -5.7e-01 | -1.4e+00 | -1.3e+00 | -8.5e-01 | 5.1e-01 | 6.0e+01 |
| n-H-B:p-C-E | 0.0e+00 | -4.0e+00 | 0.0e+00 | 0.0e+00 | 0.0e+00 | -8.1e-01 | 1.6e+00 | 2.0e+02 |
| n-C-E:p-C-B | -1.7e+00 | 0.0e+00 | -1.5e-02 | -9.7e-01 | -1.2e+00 | -7.6e-01 | 6.6e-01 | 8.6e+01 |
| n-E-B:n-C-E | -2.8e+00 | 3.2e+00 | 0.0e+00 | -3.1e+00 | -1.1e+00 | -7.5e-01 | 2.3e+00 | 3.0e+02 |
| n-C-B:p-H-B | 2.0e-01 | -5.2e-01 | -4.4e-01 | -1.5e+00 | -1.3e+00 | -7.0e-01 | 6.1e-01 | 8.6e+01 |
| p-C-E:n-H-B | 6.1e-01 | 0.0e+00 | 3.5e+00 | -4.8e+00 | -2.7e+00 | -6.9e-01 | 2.9e+00 | 4.2e+02 |
| n-C-B:p-C-B | 0.0e+00 | -2.8e+00 | 0.0e+00 | 0.0e+00 | 0.0e+00 | -5.7e-01 | 1.1e+00 | 2.0e+02 |
| n-C-B:p-E-E | 0.0e+00 | 0.0e+00 | -1.9e+00 | 0.0e+00 | 0.0e+00 | -3.9e-01 | 7.7e-01 | 2.0e+02 |
| n-E-B:n-H-B | 0.0e+00 | 0.0e+00 | -1.9e+00 | 0.0e+00 | 0.0e+00 | -3.7e-01 | 7.4e-01 | 2.0e+02 |
| p-E-B:p-C-E | 0.0e+00 | 0.0e+00 | 0.0e+00 | -1.2e+00 | 0.0e+00 | -2.3e-01 | 4.7e-01 | 2.0e+02 |
| n-H-B:p-H-B | 0.0e+00 | -1.1e+00 | 0.0e+00 | 0.0e+00 | 0.0e+00 | -2.2e-01 | 4.4e-01 | 2.0e+02 |
| n-H-B:p-H-E | 1.8e+00 | 1.8e+00 | 4.4e-01 | -2.1e+00 | -2.1e+00 | -4.8e-02 | 1.8e+00 | 3.7e+03 |
| n-H-E:p-H-B | 0.0e+00 | 3.1e-01 | 0.0e+00 | 0.0e+00 | 0.0e+00 | 6.3e-02 | 1.3e-01 | 2.0e+02 |
| p-H-B:p-C-B | 0.0e+00 | 1.1e+00 | 0.0e+00 | 0.0e+00 | 0.0e+00 | 2.2e-01 | 4.4e-01 | 2.0e+02 |
| p-H-B:p-H-E | 0.0e+00 | 1.4e+00 | 0.0e+00 | 0.0e+00 | 0.0e+00 | 2.7e-01 | 5.5e-01 | 2.0e+02 |
| p-C-B:p-H-B | 9.7e-01 | 0.0e+00 | -1.3e+00 | 1.2e+00 | 2.3e+00 | 6.4e-01 | 1.2e+00 | 1.9e+02 |
| p-H-B:p-H-B | 1.5e+00 | -3.3e+00 | 2.2e+00 | 1.2e+00 | 2.1e+00 | 7.4e-01 | 2.0e+00 | 2.7e+02 |
| n-H-B:n-H-E | 5.7e+00 | 0.0e+00 | 0.0e+00 | 0.0e+00 | 0.0e+00 | 1.1e+00 | 2.3e+00 | 2.0e+02 |
| p-C-E:p-C-B | 5.7e+00 | 0.0e+00 | 0.0e+00 | 0.0e+00 | 0.0e+00 | 1.1e+00 | 2.3e+00 | 2.0e+02 |
| n-E-B:p-H-B | 2.8e+00 | -1.5e+00 | 4.0e-01 | 1.3e+00 | 3.0e+00 | 1.2e+00 | 1.6e+00 | 1.4e+02 |
| p-C-E:n-E-B | 0.0e+00 | 0.0e+00 | 6.5e+00 | 0.0e+00 | 0.0e+00 | 1.3e+00 | 2.6e+00 | 2.0e+02 |
| n-C-B:n-E-B | 1.1e+00 | 1.8e+00 | 2.9e+00 | 1.9e+00 | 2.1e+00 | 2.0e+00 | 5.6e-01 | 2.8e+01 |
| p-E-B:p-E-B | 2.5e+00 | 3.9e+00 | -1.5e-02 | 2.8e+00 | 3.3e+00 | 2.5e+00 | 1.4e+00 | 5.4e+01 |
| p-H-B:p-E-B | 7.7e-02 | 4.8e+00 | 2.1e+00 | 4.4e+00 | 2.1e+00 | 2.7e+00 | 1.7e+00 | 6.4e+01 |
| n-H-E:p-H-E | -9.3e+00 | 3.3e+00 | 6.5e+00 | -2.6e-01 | 1.4e+01 | 2.8e+00 | 7.6e+00 | 2.7e+02 |
| p-C-B:p-E-E | 0.0e+00 | 0.0e+00 | 1.7e+01 | 0.0e+00 | 0.0e+00 | 3.3e+00 | 6.6e+00 | 2.0e+02 |
| p-H-E:p-H-B | 9.7e+00 | 0.0e+00 | -2.6e+00 | 1.8e+00 | 7.7e+00 | 3.3e+00 | 4.6e+00 | 1.4e+02 |
| n-H-E:p-E-B | 1.8e+01 | 0.0e+00 | 0.0e+00 | 0.0e+00 | 0.0e+00 | 3.6e+00 | 7.1e+00 | 2.0e+02 |
| p-C-B:p-C-E | 0.0e+00 | 9.7e+00 | -5.7e-02 | 2.5e+00 | 6.1e+00 | 3.6e+00 | 3.8e+00 | 1.0e+02 |
| n-C-E:p-C-E | -1.0e+00 | 1.3e+01 | -2.4e+00 | 4.4e-01 | 1.1e+01 | 4.2e+00 | 6.4e+00 | 1.5e+02 |
| n-C-B:p-C-E | 6.9e+00 | 4.8e+00 | 2.9e+00 | 4.0e+00 | 8.9e+00 | 5.5e+00 | 2.1e+00 | 3.8e+01 |
| p-C-E:p-H-B | 2.4e+00 | 4.4e+00 | 1.0e+01 | 1.0e+01 | 6.5e+00 | 6.8e+00 | 3.2e+00 | 4.7e+01 |
| p-C-E:n-H-E | -1.3e+01 | 2.4e+01 | 5.7e+00 | -4.4e+00 | 2.5e+01 | 7.3e+00 | 1.5e+01 | 2.0e+02 |
| p-E-E:n-E-B | 1.3e+01 | -1.9e-01 | 1.7e+00 | 1.4e+01 | 8.5e+00 | 7.5e+00 | 5.9e+00 | 7.8e+01 |
| n-C-B:n-C-E | 8.1e+00 | 4.4e+00 | 2.6e+00 | 1.3e+01 | 1.0e+01 | 7.6e+00 | 3.7e+00 | 4.8e+01 |
| n-H-E:n-C-B | 0.0e+00 | 8.1e+00 | 1.1e+01 | 8.5e+00 | 1.3e+01 | 8.2e+00 | 4.5e+00 | 5.5e+01 |
| n-E-B:p-C-E | 1.0e+01 | 4.0e+00 | 0.0e+00 | 1.6e+01 | 1.1e+01 | 8.4e+00 | 5.7e+00 | 6.8e+01 |
| n-H-B:n-E-E | 6.1e+01 | 7.7e+00 | -3.3e+01 | 3.4e+01 | -2.1e+01 | 9.6e+00 | 3.5e+01 | 3.6e+02 |
| n-H-E:n-H-B | 0.0e+00 | 1.3e+01 | 6.9e+00 | 2.3e+01 | 7.3e+00 | 9.8e+00 | 7.5e+00 | 7.6e+01 |
| n-C-B:p-H-E | 1.5e+01 | 1.1e+01 | 4.4e+00 | 5.7e+00 | 1.6e+01 | 1.1e+01 | 4.8e+00 | 4.6e+01 |
| p-E-E:n-E-E | 0.0e+00 | 0.0e+00 | 0.0e+00 | 0.0e+00 | 6.1e+01 | 1.2e+01 | 2.4e+01 | 2.0e+02 |
| p-H-E:p-E-B | 2.3e+00 | 3.2e+01 | 1.3e+01 | 5.7e+00 | 1.1e+01 | 1.3e+01 | 1.0e+01 | 8.0e+01 |
| n-E-E:p-E-B | 1.3e+01 | 1.7e+01 | 1.7e+01 | -2.4e-01 | 2.4e+01 | 1.4e+01 | 8.1e+00 | 5.6e+01 |
| p-E-B:n-H-E | 0.0e+00 | 7.3e+00 | 1.0e+00 | 3.3e+01 | 3.6e+01 | 1.6e+01 | 1.6e+01 | 1.0e+02 |
| n-E-E:p-E-E | -3.3e+01 | 7.7e+01 | -6.5e+01 | 1.0e+02 | 0.0e+00 | 1.7e+01 | 6.4e+01 | 3.8e+02 |
| p-H-E:p-C-E | 2.1e+01 | 2.3e+01 | 6.9e+00 | 2.1e+01 | 1.5e+01 | 1.7e+01 | 6.0e+00 | 3.4e+01 |
| n-E-E:p-H-B | 0.0e+00 | 0.0e+00 | 0.0e+00 | 0.0e+00 | 9.7e+01 | 1.9e+01 | 3.9e+01 | 2.0e+02 |
| n-C-E:n-C-E | 2.3e+01 | 1.7e+01 | 3.8e+01 | 1.7e+01 | 1.7e+01 | 2.2e+01 | 8.2e+00 | 3.7e+01 |
| p-H-E:p-E-E | 0.0e+00 | 1.2e+02 | 0.0e+00 | 0.0e+00 | 0.0e+00 | 2.3e+01 | 4.7e+01 | 2.0e+02 |
| n-C-B:n-E-E | 4.4e+00 | 5.2e+01 | 8.9e+00 | 2.9e+01 | 3.4e+01 | 2.6e+01 | 1.8e+01 | 6.8e+01 |
| n-H-E:n-E-B | 3.6e+01 | 4.8e+01 | 1.9e+01 | 2.2e+01 | 7.7e+00 | 2.7e+01 | 1.4e+01 | 5.3e+01 |
| p-H-B:n-E-E | 4.8e+01 | 6.5e+01 | 7.7e+01 | 4.4e+01 | 0.0e+00 | 4.7e+01 | 2.6e+01 | 5.6e+01 |
| n-E-E:p-C-E | 7.3e+01 | 2.5e+01 | 6.9e+01 | 8.9e+01 | 3.4e+01 | 5.8e+01 | 2.4e+01 | 4.2e+01 |
| n-C-E:n-E-E | 8.1e+01 | 3.2e+00 | 1.2e+02 | 1.2e+02 | -1.7e+01 | 6.1e+01 | 5.7e+01 | 9.4e+01 |
| p-E-E:p-H-E | 2.4e+02 | 0.0e+00 | 2.7e+02 | 2.9e+02 | 9.7e+01 | 1.8e+02 | 1.1e+02 | 6.3e+01 |
| n-H-E:n-E-E | -4.8e+02 | 8.1e+01 | 1.0e+03 | -1.4e+02 | 1.2e+03 | 3.3e+02 | 6.5e+02 | 2.0e+02 |
| ϕ parameter *min* | 1-fold | 2-fold | 3-fold | 4-fold | 5-fold | Average | RMSD | % RMSD |
| n-E-E:n-E-E | -1.3e+02 | -1.4e+02 | -2.5e+02 | -1.9e+02 | -3.3e+02 | -2.1e+02 | 7.4e+01 | 3.6e+01 |
| p-E-E:p-E-E | -4.4e+01 | -8.8e+01 | -1.2e+02 | -2.2e+02 | -9.9e+01 | -1.1e+02 | 5.8e+01 | 5.1e+01 |
| n-E-B:p-H-E | 0.0e+00 | -1.2e+02 | -6.9e+01 | -1.2e+02 | -1.6e+02 | -9.4e+01 | 5.5e+01 | 5.9e+01 |
| n-H-E:n-H-E | -9.4e+01 | -1.6e+01 | -1.3e+02 | -9.9e+01 | -8.3e+01 | -8.4e+01 | 3.8e+01 | 4.5e+01 |
| n-H-B:p-E-E | -7.2e+01 | 8.6e+00 | -8.0e+01 | -1.3e+02 | -1.3e+02 | -8.1e+01 | 5.1e+01 | 6.3e+01 |
| p-C-E:p-E-E | -5.9e+01 | -8.7e+01 | -1.0e+02 | -2.3e+01 | -7.9e+01 | -7.0e+01 | 2.7e+01 | 3.9e+01 |
| n-E-E:n-C-E | -9.9e+01 | -5.5e+01 | -1.1e+02 | 0.0e+00 | -5.5e+01 | -6.4e+01 | 3.9e+01 | 6.1e+01 |
| p-H-E:p-E-B | -7.8e+01 | -8.5e+00 | -6.2e+01 | -3.6e+01 | -1.3e+02 | -6.3e+01 | 4.1e+01 | 6.5e+01 |
| p-C-E:n-E-E | -5.9e+01 | -1.8e+02 | 0.0e+00 | -1.1e+01 | -5.6e+01 | -6.1e+01 | 6.4e+01 | 1.0e+02 |
| p-H-E:n-C-E | 0.0e+00 | -7.8e+01 | -7.0e+01 | -4.7e+01 | -7.3e+01 | -5.4e+01 | 2.9e+01 | 5.4e+01 |
| p-H-B:n-E-E | 0.0e+00 | 3.3e+01 | -1.2e+02 | -3.2e+01 | -1.2e+02 | -4.8e+01 | 6.2e+01 | 1.3e+02 |
| n-H-B:n-H-E | -3.3e+01 | -4.7e+01 | -4.0e+01 | -1.9e+01 | -2.9e+01 | -3.4e+01 | 9.5e+00 | 2.8e+01 |
| n-H-E:n-C-E | 0.0e+00 | 0.0e+00 | -1.5e+02 | 0.0e+00 | 0.0e+00 | -3.0e+01 | 6.0e+01 | 2.0e+02 |
| p-H-E:p-C-E | 1.4e+01 | -2.8e+01 | -7.5e+01 | -5.9e+01 | 0.0e+00 | -3.0e+01 | 3.4e+01 | 1.1e+02 |
| n-C-B:n-E-E | -1.9e+01 | 3.7e+01 | -2.5e+01 | -3.0e+01 | -1.0e+02 | -2.7e+01 | 4.4e+01 | 1.6e+02 |
| p-E-E:p-E-B | -3.0e+00 | 3.5e+00 | -5.1e+01 | -4.3e+01 | -3.8e+01 | -2.6e+01 | 2.2e+01 | 8.4e+01 |
| p-E-E:n-C-B | -1.3e+01 | -4.0e+01 | -2.4e+01 | -1.2e+01 | -6.7e+00 | -1.9e+01 | 1.2e+01 | 6.2e+01 |
| p-E-E:p-H-B | 1.8e+01 | 0.0e+00 | -3.7e+01 | -2.1e+01 | -5.2e+01 | -1.8e+01 | 2.5e+01 | 1.4e+02 |
| n-E-E:p-H-B | -8.6e+01 | 0.0e+00 | 0.0e+00 | 0.0e+00 | 0.0e+00 | -1.7e+01 | 3.4e+01 | 2.0e+02 |
| p-H-E:n-E-B | -6.9e+01 | 0.0e+00 | 0.0e+00 | 0.0e+00 | 0.0e+00 | -1.4e+01 | 2.8e+01 | 2.0e+02 |
| n-C-E:p-H-E | -6.2e+01 | 0.0e+00 | 0.0e+00 | 0.0e+00 | 0.0e+00 | -1.2e+01 | 2.5e+01 | 2.0e+02 |
| p-H-B:p-H-E | -8.7e+00 | -1.5e+01 | -8.6e+00 | -1.6e+01 | -7.6e+00 | -1.1e+01 | 3.6e+00 | 3.2e+01 |
| p-C-B:p-E-E | 0.0e+00 | 4.3e+01 | -5.9e+01 | 2.9e+01 | -6.4e+01 | -1.0e+01 | 4.4e+01 | 4.3e+02 |
| n-H-B:p-E-B | -7.5e+00 | -4.0e+00 | 0.0e+00 | -2.0e+01 | -1.3e+01 | -8.9e+00 | 7.0e+00 | 7.9e+01 |
| p-C-E:p-H-E | 0.0e+00 | 0.0e+00 | 0.0e+00 | 0.0e+00 | -4.4e+01 | -8.8e+00 | 1.8e+01 | 2.0e+02 |
| n-H-E:n-C-B | 4.4e+01 | 2.5e+01 | -1.8e+01 | -6.9e+01 | -1.1e+01 | -5.8e+00 | 3.9e+01 | 6.7e+02 |
| p-H-B:p-E-B | 7.9e-01 | 3.2e+00 | -1.4e+01 | -1.1e+01 | -7.7e+00 | -5.7e+00 | 6.7e+00 | 1.2e+02 |
| n-E-B:p-H-B | -5.3e+00 | -7.8e+00 | -1.5e+00 | -6.0e+00 | -2.7e+00 | -4.7e+00 | 2.3e+00 | 4.9e+01 |
| n-E-E:p-E-E | 0.0e+00 | 0.0e+00 | -2.0e+01 | 0.0e+00 | 0.0e+00 | -4.0e+00 | 8.0e+00 | 2.0e+02 |
| n-H-E:n-E-B | -1.3e+02 | 5.2e+02 | -1.5e+02 | -1.0e+02 | -1.6e+02 | -4.0e+00 | 2.6e+02 | 6.6e+03 |
| n-C-B:p-H-B | -6.5e+00 | -4.8e+00 | -1.5e+00 | -2.0e+00 | -2.8e+00 | -3.5e+00 | 1.9e+00 | 5.3e+01 |
| p-E-E:n-E-B | 1.2e+01 | -1.0e+01 | -8.1e+00 | -1.5e+01 | 8.3e+00 | -2.6e+00 | 1.1e+01 | 4.2e+02 |
| n-C-B:p-C-E | 0.0e+00 | -4.1e+00 | -1.1e+01 | 1.5e+00 | 1.7e+00 | -2.4e+00 | 4.8e+00 | 2.0e+02 |
| n-E-E:p-C-E | 0.0e+00 | 0.0e+00 | -1.1e+01 | 0.0e+00 | 0.0e+00 | -2.2e+00 | 4.4e+00 | 2.0e+02 |
| p-H-E:p-C-B | 0.0e+00 | 0.0e+00 | -9.2e+00 | 0.0e+00 | 0.0e+00 | -1.8e+00 | 3.7e+00 | 2.0e+02 |
| n-C-B:n-E-B | -3.7e+00 | -2.0e+00 | 0.0e+00 | -1.2e+00 | -7.9e-01 | -1.5e+00 | 1.3e+00 | 8.2e+01 |
| p-E-B:n-H-B | 0.0e+00 | 0.0e+00 | -7.7e+00 | 0.0e+00 | 0.0e+00 | -1.5e+00 | 3.1e+00 | 2.0e+02 |
| n-H-B:p-H-B | -3.0e-01 | 0.0e+00 | -2.5e+00 | -6.6e-01 | -1.6e+00 | -1.0e+00 | 9.2e-01 | 9.1e+01 |
| p-C-E:n-C-B | -5.1e+00 | 0.0e+00 | 0.0e+00 | 0.0e+00 | 0.0e+00 | -1.0e+00 | 2.0e+00 | 2.0e+02 |
| n-E-B:n-C-E | 0.0e+00 | 0.0e+00 | 0.0e+00 | 0.0e+00 | -4.3e+00 | -8.6e-01 | 1.7e+00 | 2.0e+02 |
| n-H-B:n-E-B | 1.6e+00 | -2.1e-01 | 0.0e+00 | -5.6e+00 | 1.1e+00 | -6.2e-01 | 2.6e+00 | 4.1e+02 |
| p-H-B:p-H-B | -3.2e+00 | -6.1e-01 | -2.3e+00 | 2.4e+00 | 5.9e-01 | -6.2e-01 | 2.0e+00 | 3.2e+02 |
| n-H-B:n-C-B | 9.0e-01 | 0.0e+00 | -4.8e-01 | -8.6e-01 | -1.5e+00 | -3.9e-01 | 8.1e-01 | 2.1e+02 |
| n-H-B:n-H-B | -3.3e-01 | -7.1e-01 | -2.8e-01 | 7.0e-01 | -1.1e+00 | -3.4e-01 | 6.0e-01 | 1.7e+02 |
| p-C-B:p-E-B | -4.3e+00 | -6.7e-01 | 9.1e-01 | 2.4e+00 | 3.3e-01 | -2.7e-01 | 2.2e+00 | 8.5e+02 |
| p-H-B:n-H-B | 0.0e+00 | 3.5e-01 | 0.0e+00 | 0.0e+00 | 0.0e+00 | 7.0e-02 | 1.4e-01 | 2.0e+02 |
| p-C-E:p-E-B | 2.8e+00 | -5.3e+00 | 7.7e+00 | -6.0e+00 | 1.5e+00 | 1.4e-01 | 5.2e+00 | 3.7e+03 |
| n-E-B:n-H-B | 0.0e+00 | 0.0e+00 | 8.9e-01 | 0.0e+00 | 0.0e+00 | 1.8e-01 | 3.6e-01 | 2.0e+02 |
| n-E-B:n-C-B | 0.0e+00 | 0.0e+00 | 1.2e+00 | 0.0e+00 | 0.0e+00 | 2.4e-01 | 4.8e-01 | 2.0e+02 |
| n-C-E:n-E-B | 5.8e+00 | 1.1e+01 | 4.5e+00 | -2.0e+01 | 0.0e+00 | 2.6e-01 | 1.1e+01 | 4.1e+03 |
| n-C-B:n-H-B | 0.0e+00 | 2.8e+00 | 0.0e+00 | 0.0e+00 | 0.0e+00 | 5.6e-01 | 1.1e+00 | 2.0e+02 |
| p-C-B:p-H-B | 1.2e+00 | 5.3e-01 | 2.5e-01 | -2.7e-01 | 1.9e+00 | 7.2e-01 | 7.6e-01 | 1.0e+02 |
| p-C-B:n-E-B | 0.0e+00 | 0.0e+00 | 4.8e+00 | 0.0e+00 | 0.0e+00 | 9.6e-01 | 1.9e+00 | 2.0e+02 |
| p-C-E:n-H-B | 0.0e+00 | 0.0e+00 | 0.0e+00 | 5.4e+00 | 0.0e+00 | 1.1e+00 | 2.2e+00 | 2.0e+02 |
| n-C-B:p-C-B | 0.0e+00 | 7.9e+00 | 0.0e+00 | 0.0e+00 | 0.0e+00 | 1.6e+00 | 3.2e+00 | 2.0e+02 |
| p-H-B:p-E-E | 0.0e+00 | 1.1e+01 | 0.0e+00 | 0.0e+00 | 0.0e+00 | 2.2e+00 | 4.4e+00 | 2.0e+02 |
| p-C-B:n-H-B | -1.4e-01 | 1.1e+00 | 3.7e+00 | 6.6e+00 | 3.6e+00 | 3.0e+00 | 2.3e+00 | 7.9e+01 |
| n-C-B:p-H-E | -9.2e-02 | 7.7e+00 | 1.5e+01 | -2.8e+00 | 5.2e-02 | 4.0e+00 | 6.5e+00 | 1.6e+02 |
| n-E-B:p-C-B | 5.7e+00 | 5.7e+00 | 0.0e+00 | 2.5e+00 | 7.9e+00 | 4.4e+00 | 2.8e+00 | 6.4e+01 |
| p-E-B:p-E-B | 2.3e+00 | 8.3e+00 | 5.4e+00 | 6.1e+00 | 3.9e+00 | 5.2e+00 | 2.0e+00 | 3.9e+01 |
| p-C-E:p-H-B | 0.0e+00 | 2.9e+01 | 0.0e+00 | 0.0e+00 | 0.0e+00 | 5.8e+00 | 1.2e+01 | 2.0e+02 |
| n-C-B:n-C-B | 3.0e+00 | -6.9e-02 | 1.2e+01 | 6.4e+00 | 1.0e+01 | 6.3e+00 | 4.4e+00 | 7.1e+01 |
| n-C-E:p-C-B | 3.2e+01 | 0.0e+00 | 0.0e+00 | 0.0e+00 | 0.0e+00 | 6.4e+00 | 1.3e+01 | 2.0e+02 |
| p-C-B:n-C-B | 4.6e+00 | 0.0e+00 | 8.3e+00 | 9.5e+00 | 9.6e+00 | 6.4e+00 | 3.7e+00 | 5.7e+01 |
| n-C-B:p-E-B | 7.4e-01 | 2.5e+00 | 8.1e+00 | 1.1e+01 | 1.0e+01 | 6.5e+00 | 4.1e+00 | 6.3e+01 |
| p-C-B:p-C-E | 6.7e+00 | 6.7e+00 | 6.5e+00 | 1.5e+01 | 2.8e-02 | 7.0e+00 | 4.8e+00 | 6.8e+01 |
| n-C-B:n-C-E | 7.8e+00 | 8.8e+00 | 1.5e+01 | 5.0e-02 | 4.1e+00 | 7.2e+00 | 5.0e+00 | 7.0e+01 |
| n-H-B:p-H-E | 3.7e+00 | 9.0e+00 | 3.1e+00 | 9.7e+00 | 1.2e+01 | 7.5e+00 | 3.5e+00 | 4.7e+01 |
| p-C-B:p-H-E | -6.3e+00 | 2.9e+01 | 0.0e+00 | 1.1e+00 | 1.4e+01 | 7.6e+00 | 1.3e+01 | 1.7e+02 |
| p-E-B:n-E-E | 0.0e+00 | 0.0e+00 | 4.1e+01 | 0.0e+00 | 0.0e+00 | 8.2e+00 | 1.6e+01 | 2.0e+02 |
| n-C-E:n-H-B | 2.4e+00 | -5.7e-01 | 1.8e+01 | 1.4e+01 | 8.9e+00 | 8.5e+00 | 6.9e+00 | 8.1e+01 |
| n-E-B:n-E-B | 8.7e+00 | 9.3e+00 | 7.5e+00 | 1.0e+01 | 9.0e+00 | 8.9e+00 | 8.2e-01 | 9.2e+00 |
| n-C-E:p-H-B | 1.2e+01 | 5.8e+00 | 1.6e+01 | 4.7e+00 | 7.7e+00 | 9.2e+00 | 4.2e+00 | 4.5e+01 |
| p-H-E:n-H-E | 0.0e+00 | 0.0e+00 | 0.0e+00 | 0.0e+00 | 4.6e+01 | 9.2e+00 | 1.8e+01 | 2.0e+02 |
| p-C-E:p-C-E | 1.8e+01 | -2.4e+01 | 3.3e+01 | 2.1e+01 | 4.0e+00 | 1.0e+01 | 2.0e+01 | 1.9e+02 |
| n-H-B:p-C-E | 8.4e+00 | 8.9e+00 | 1.9e+01 | 0.0e+00 | 1.9e+01 | 1.1e+01 | 7.2e+00 | 6.5e+01 |
| p-E-E:p-C-B | 5.5e+01 | 0.0e+00 | 0.0e+00 | 0.0e+00 | 0.0e+00 | 1.1e+01 | 2.2e+01 | 2.0e+02 |
| n-E-B:p-E-B | 9.5e+00 | 9.8e+00 | 1.3e+01 | 1.2e+01 | 1.5e+01 | 1.2e+01 | 2.0e+00 | 1.7e+01 |
| p-C-B:p-C-B | 7.3e+00 | 6.5e+00 | 1.2e+01 | 1.8e+01 | 1.6e+01 | 1.2e+01 | 4.6e+00 | 3.8e+01 |
| p-C-E:n-E-B | 0.0e+00 | 0.0e+00 | 5.8e+01 | 0.0e+00 | 0.0e+00 | 1.2e+01 | 2.3e+01 | 2.0e+02 |
| p-E-B:n-C-E | 2.3e+01 | 1.4e+01 | 2.1e+01 | 2.2e+01 | 1.0e+00 | 1.6e+01 | 8.2e+00 | 5.1e+01 |
| n-E-B:n-E-E | 5.6e+01 | 5.5e+01 | -3.2e+00 | -2.4e+01 | 1.0e+00 | 1.7e+01 | 3.3e+01 | 1.9e+02 |
| p-C-B:n-C-E | 0.0e+00 | 2.8e+01 | 2.5e+01 | 1.3e+01 | 1.8e+01 | 1.7e+01 | 9.9e+00 | 5.9e+01 |
| p-H-B:p-C-E | 2.8e+01 | 0.0e+00 | 2.7e+01 | 1.5e+01 | 1.6e+01 | 1.7e+01 | 1.0e+01 | 5.9e+01 |
| n-C-E:n-E-E | 0.0e+00 | 0.0e+00 | 0.0e+00 | 1.1e+02 | 0.0e+00 | 2.2e+01 | 4.4e+01 | 2.0e+02 |
| p-H-B:n-H-E | 1.7e+01 | -1.9e+00 | 4.0e+01 | 4.1e+01 | 2.0e+01 | 2.3e+01 | 1.6e+01 | 6.9e+01 |
| n-C-E:n-C-E | 5.3e+01 | 2.5e+00 | -9.9e-01 | 4.2e+01 | 2.1e+01 | 2.4e+01 | 2.1e+01 | 9.0e+01 |
| n-E-E:p-E-B | 8.0e+01 | 4.2e+01 | 0.0e+00 | -6.5e+00 | 7.5e+00 | 2.5e+01 | 3.2e+01 | 1.3e+02 |
| n-C-E:n-H-E | 2.7e+01 | 8.6e+01 | 0.0e+00 | 1.2e+01 | 1.3e+01 | 2.8e+01 | 3.0e+01 | 1.1e+02 |
| n-H-B:n-E-E | 0.0e+00 | 0.0e+00 | 1.4e+02 | 0.0e+00 | 0.0e+00 | 2.8e+01 | 5.6e+01 | 2.0e+02 |
| n-H-E:p-H-E | 6.0e+01 | 4.8e+01 | 5.6e-01 | 3.8e+01 | 0.0e+00 | 2.9e+01 | 2.5e+01 | 8.4e+01 |
| n-C-E:p-C-E | 1.1e+01 | 2.8e+01 | 2.6e+01 | 5.5e+01 | 3.2e+01 | 3.0e+01 | 1.4e+01 | 4.7e+01 |
| n-E-B:p-C-E | 3.5e+01 | 5.1e+01 | 0.0e+00 | 3.8e+01 | 3.5e+01 | 3.2e+01 | 1.7e+01 | 5.3e+01 |
| n-E-E:p-C-B | 0.0e+00 | 0.0e+00 | 1.7e+02 | 0.0e+00 | 0.0e+00 | 3.4e+01 | 6.8e+01 | 2.0e+02 |
| p-H-E:p-H-E | 9.7e+01 | 3.9e+01 | 1.9e+01 | 2.8e+01 | 3.2e+01 | 4.3e+01 | 2.8e+01 | 6.5e+01 |
| p-C-B:n-E-E | 5.9e+01 | -7.4e-02 | 0.0e+00 | 1.0e+02 | 1.6e+02 | 6.4e+01 | 6.1e+01 | 9.6e+01 |
| p-C-E:n-H-E | 8.4e+01 | 1.0e+02 | 5.9e+01 | -3.3e+01 | 1.8e+02 | 7.8e+01 | 6.9e+01 | 8.8e+01 |
| n-E-E:n-H-B | 9.1e+01 | 9.4e+00 | 0.0e+00 | 2.5e+02 | 8.1e+01 | 8.6e+01 | 9.0e+01 | 1.0e+02 |
| n-H-E:p-C-B | 7.0e+01 | 6.4e+01 | 9.2e+01 | 1.1e+02 | 1.2e+02 | 9.1e+01 | 2.2e+01 | 2.4e+01 |
| p-E-E:n-E-E | 8.2e+01 | 1.3e+02 | 0.0e+00 | 1.1e+02 | 1.7e+02 | 9.8e+01 | 5.7e+01 | 5.8e+01 |
| n-C-E:p-E-E | 1.6e+02 | 1.9e+02 | 2.8e+02 | 5.6e+01 | 2.7e+02 | 1.9e+02 | 8.2e+01 | 4.3e+01 |
| n-H-E:p-E-B | -4.4e+01 | 0.0e+00 | 1.6e+03 | 1.5e+03 | 1.6e+03 | 9.3e+02 | 7.8e+02 | 8.4e+01 |
| p-E-B:n-H-E | 0.0e+00 | 7.3e+03 | 0.0e+00 | 0.0e+00 | 0.0e+00 | 1.5e+03 | 2.9e+03 | 2.0e+02 |
| n-E-E:n-E-E | -1.3e+02 | -1.4e+02 | -2.5e+02 | -1.9e+02 | -3.3e+02 | -2.1e+02 | 7.4e+01 | 3.6e+01 |
| p-E-E:p-E-E | -4.4e+01 | -8.8e+01 | -1.2e+02 | -2.2e+02 | -9.9e+01 | -1.1e+02 | 5.8e+01 | 5.1e+01 |
| n-E-B:p-H-E | 0.0e+00 | -1.2e+02 | -6.9e+01 | -1.2e+02 | -1.6e+02 | -9.4e+01 | 5.5e+01 | 5.9e+01 |
